# Identifying patterns of dispersal, connectivity and selection in the sea scallop, *Placopecten magellanicus,* using RADseq‐derived SNPs

**DOI:** 10.1111/eva.12432

**Published:** 2016-11-02

**Authors:** Mallory Van Wyngaarden, Paul V. R. Snelgrove, Claudio DiBacco, Lorraine C. Hamilton, Naiara Rodríguez‐Ezpeleta, Nicholas W. Jeffery, Ryan R. E. Stanley, Ian R. Bradbury

**Affiliations:** ^1^ Department of BiologyMemorial University of NewfoundlandSt. John'sNLCanada; ^2^ Department of Ocean SciencesMemorial University of NewfoundlandSt. John'sNLCanada; ^3^ Bedford Institute of OceanographyDartmouthNSCanada; ^4^ Aquatic Biotechnology LabBedford Institute of OceanographyDartmouthNSCanada; ^5^ Marine Research DivisionAZTI TechnaliaSukarrietaBizkaiaSpain; ^6^ Fisheries and Oceans CanadaNorthwest Atlantic Fisheries CentreSt. John'sNLCanada; ^7^ Faculty of Computer ScienceDalhousie UniversityHalifaxNSCanada

**Keywords:** connectivity, dispersal, outlier loci, population genomics, population structure, RADseq, sea scallop, single nucleotide polymorphism

## Abstract

Understanding patterns of dispersal and connectivity among marine populations can directly inform fisheries conservation and management. Advances in high‐throughput sequencing offer new opportunities for estimating marine connectivity. We used restriction‐site‐associated DNA sequencing to examine dispersal and realized connectivity in the sea scallop *Placopecten magellanicus*, an economically important marine bivalve. Based on 245 individuals sampled rangewide at 12 locations from Newfoundland to the Mid‐Atlantic Bight, we identified and genotyped 7163 single nucleotide polymorphisms; 112 (1.6%) were identified as outliers potentially under directional selection. Bayesian clustering revealed a discontinuity between northern and southern samples, and latitudinal clines in allele frequencies were observed in 42.9% of the outlier loci and in 24.6% of neutral loci. Dispersal estimates derived using these clines and estimates of linkage disequilibrium imply limited dispersal; 373.1 ± 407.0 km (mean ± SD) for outlier loci and 641.0 ± 544.6 km (mean ± SD) for neutral loci. Our analysis suggests restricted dispersal compared to the species range (>2000 km) and that dispersal and effective connectivity differ. These observations support the hypothesis that limited effective dispersal structures scallop populations along eastern North America. These findings can help refine the appropriate scale of management and conservation in this commercially valuable species.

## 1 Introduction

Successful species management and conservation require an accurate understanding of population connectivity, including interbreeding and dispersal among populations (Allendorf, Hohenlohe, & Luikart, 2010). The degree of connectivity among adjacent populations can affect population persistence, productivity and response to exploitation (Cowen, Paris, & Srinivasan, 2006; Gaines, Gaylord, & Largier, 2003; Hastings & Botsford, 2006; Hellberg, Burton, Neigel, & Palumbi, 2002; Lowe & Allendorf, 2010; Palumbi, 2003; Waples, 1998). In terrestrial systems, connectivity quantification methods include individual tracking and mark–recapture, but many factors unique to the marine environment and to marine organisms complicate measurements of marine connectivity. In many cases, the large effective sizes of temperate marine populations prevent genetic drift from promoting differentiation over short‐to‐moderate timescales, limiting the accumulation of neutral genomic divergence (Hauser & Carvalho, 2008). In addition, most marine invertebrates, especially sessile, benthic species, reproduce via broadcast spawning; high larval dispersal potential characterizes these types of organisms (Cowen & Sponaugle, 2009; Hauser & Carvalho, 2008; Neilsen & Kenchington, 2001) potentially producing a mixed pool of larvae from different populations (Thorrold et al., 2002) and contributing to the assumption of limited marine population structure (Cowen, Lwiza, Sponaugle, Paris, & Olson, 2000). However, over the last few decades, accumulating phenotypic and genotypic evidence suggests limited dispersal and low connectivity drive fine‐scale population structure that may be more common than previously expected in marine environments (Hauser & Carvalho, 2008; Hellberg, 2009), potentially challenging current management paradigms in many exploited marine species.

Advances in genetic and genomic techniques drive much of the emerging evidence of limited dispersal and connectivity in marine species (Benestan et al., 2015; Bradbury & Bentzen, 2007; Catchen et al., 2013; Hedgecock, Barber, & Edmands, 2007; Kinlan & Gaines, 2003; Milano et al., 2014; Reitzel, Herrera, Layden, Martindale, & Shank, 2013; Sotka & Palumbi, 2006). In particular, high‐throughput, next‐generation sequencing techniques have dramatically increased the number and type of genetic loci available to study in marine species, particularly nonmodel species. The ability to survey genomewide diversity and target loci potentially associated with adaptive variation has proven particularly informative in large marine populations where directional selection may drive rapid divergence and differentiation (Allendorf et al., 2010; Bradbury et al., 2010; Gagnaire et al., 2015; Hauser & Carvalho, 2008; Jones, Srinivasan, & Almany, 2007). Examination of outlier loci (those potentially under selection) consistently demonstrates small‐scale genetic differentiation in a variety of marine taxa including *Haliotis rufescens* (red abalone) (De Wit & Palumbi, 2013), *Clupea harengus (*Atlantic herring) (Lamichhaney et al., 2012) and *Gadus morhua* (Atlantic cod) (Bradbury et al., 2010). The advent of restriction‐site‐associated DNA sequencing (RADseq) (Baird et al., 2008; Miller, Dunham, Amores, Cresko, & Johnson, 2007) now permits genomewide scans for outlier loci in model and nonmodel organisms and increases the characterization of genetic diversity and differentiation in marine species from fishes (Catchen et al., 2013; Hohenlohe et al., 2010) to invertebrates (Benestan et al., 2015; Reitzel et al., 2013).

*Placopecten magellanicus* (Gmelin) (sea scallop), a dioecious bivalve, inhabits benthic environments in the Northwest Atlantic Ocean from Newfoundland, Canada in the north to Cape Hatteras, North Carolina, USA in the south (Posgay, 1957). Sea scallops typically occur along the continental shelf at depths from approximately 10–100 m but as deep as 384 m (Naidu & Robert, 2006). The sea scallop fishery extends back over 100 years and currently represents one of the most economically important fisheries in North America in landed value on the east coast of the United States and Canada (Naidu & Robert, 2006), in 2014 comprising 7.4% of the total landing value for all Atlantic coast fisheries in Canada (4th most valuable fishery) (DFO 2016) and 7.7% of the total landed value in the United States (NOAA 2016). High fecundity, broadcast spawning and a long planktonic larval period (30–35 days) all contribute to long‐distance dispersal potential among sea scallop populations (Naidu & Robert, 2006). Despite this high potential for population interconnectivity, past studies report phenotypic differences among sea scallop populations over fine‐to‐moderate spatial scales, including differences in reproductive timing (Naidu, 1970), population‐specific fecundity (Barber, Getchell, Shumway, & Schick, 1988), shell morphometry (Kenchington & Full, 1994), larval behaviour (Manuel, Gallager, Pearce, Manning, & Odor, 1996) and growth (Naidu & Robert, 2006). Ultimately, the scale of dispersal and connectivity in this species remains unresolved and this knowledge could directly inform fisheries management and conservation efforts.

The objective of this study was to investigate sea scallop spatial population structure in the Northwest Atlantic using RADseq‐derived single nucleotide polymorphisms (SNPs), the first rangewide genomic study in this economically important species and one of only a few RADseq studies in marine bivalves. We hypothesized that previously unidentified range‐scale population structure exists in the sea scallop and that the combined use of genomewide neutral and outlier markers would provide a more powerful tool to detect finer structure than previous studies. The specific objectives were to (i) describe the spatial population structure of sea scallop in the Northwest Atlantic using RADseq‐derived SNPs, (ii) contrast the structure present at multiple spatial scales and with outlier and nonoutlier loci and (iii) estimate average dispersal distances among populations using the isolation by distance (IBD) relationship and clines in allele frequency. This work builds directly on previous scallop studies using both microsatellites (Kenchington, Patwary, Zouros, & Bird, 2006) and AFLPs (Owen & Rawson, 2013) to explore population structure and oceanographic influences in this region. It also builds on past work in Northwest Atlantic cod which reported latitudinal clines in allele frequency in outlier loci (Bradbury et al., 2010, 2013, 2014). Finally, it contributes to the spatial management of exploited stocks through the genetic characterization of populations, an important concern in this cross‐border species, as well as providing information for the identification of potential adaptive diversity (Shafer et al., 2015) which may contribute to effective management decisions in future.

## 2 Methods

### 2.1 Sample collection

**Figure 1:**
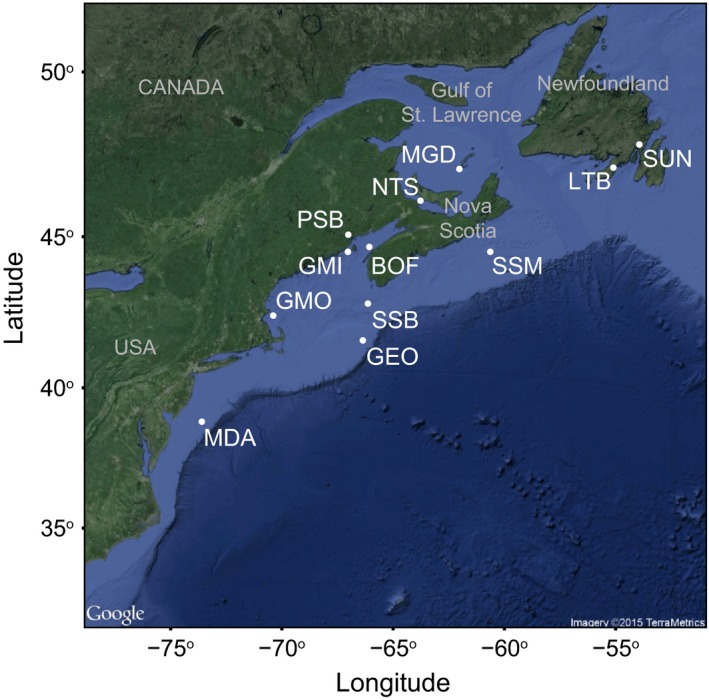
Map of 12 sea scallop (*P. magellanicus*) collection locations from the Northwest Atlantic. Site MDA (Mid‐Atlantic Bight) represents the middle of several nearby collection locations grouped as one population

We collected 252 adult scallops by hand or bottom trawl from a total of 12 locations across the entire range of the species between 2011 and 2013 (Table 1, Figure 1). Tissue samples were collected and preserved in AllProtect (Qiagen, Toronto, ON, Canada) or 80% ethanol. DNA extraction and RADseq library preparation were performed by the Aquatic Biotechnology Lab at the Bedford Institute of Oceanography in Halifax, Nova Scotia. DNA was isolated from the tissue samples using DNeasy Blood and Tissue kit or DNeasy 96 Blood and Tissue kit (Qiagen) following the manufacturer's protocol, including the optional RNase A treatment. All DNA samples were quantified using the Qubit dsDNA HS Assay Kit (Life Technologies, Burlington, ON, Canada) with assays read on a Qubit v2.0 (Life Technologies) or using the Quant‐iT PicoGreen dsDNA Assay Kit (Life Technologies) with assays read on a FLUOStar OPTIMA fluorescence plate reader (BMG Labtech, Ortenberg, Germany). The DNA quality for all samples was verified by agarose gel electrophoresis of 100 ng of extracted DNA, visualized using SYBR Safe (Life Technologies) and documented using a Gel Logic 200 (Kodak).

**Table 1:** Site name, site code, coordinates, and the number of sequenced *P. magellanicus* from each of 12 collection sites in the Northwest Atlantic Ocean

Site Name	Site Code	Latitude	Longitude	Number of scallops used in analysis
Sunnyside, NL	SUN	47.8241	−53.8695	20
Little Bay, NL	LTB	47.1545	−55.1042	21
Magdalen Islands	MGD	47.1143	−62.0243	21
Northumberland Strait	NTS	46.1338	−63.7728	22
Passamaquoddy Bay	PSB	45.0647	−67.0166	12
Bay of Fundy	BOF	44.6762	−66.0718	22
Scotian Shelf ‐ Middle	SSM	44.5207	−60.6350	19
Gulf of Maine Inshore	GMI	44.5200	−67.0319	20
Browns Bank	SSB	42.8372	−66.1358	22
Gulf of Maine Offshore	GMO	42.4400	−70.3874	22
George's Bank	GEO	41.6127	−66.3622	22
Mid Atlantic Bight^a^	MDA	38.8227	−73.5990	22

a Several neighbouring sites sampled as one location.

### 2.2 RADseq analysis

One μg of DNA was used per individual for library preparation and sequencing. RADseq libraries were prepared using *Sbf1* as described by Etter, Preston, Bassham, Cresko, and Johnson (2011) [see also Etter, Bassham, Hohenlohe, Johnson, and Cresko (2011)] with modifications. DNA samples from 22 individuals from the same geographic location comprised each library (with the exception of the library for SUN which consisted of only 20 individuals) with a different in‐line barcode in the P1 adapter for each individual sample. With the exception of SSB, GEO and SUN, the P1 adapter in‐line barcodes were all 6 bp in length. For the SSB, GEO and SUN libraries, the P1 adapter in‐line barcodes all ranged from 5 bp to 9 bp in length and were chosen to ensure equal distribution of all nucleotides at each base position (including those that overlap with the restriction site) and to maximize the edit distance (Faircloth & Glenn, 2012). Based on edit tags analysis (Faircloth & Glenn, 2012), the variable length barcodes edit distance ranged from 2 to 8 with a modal edit distance of 6. Gel size selection after sonication and PCR amplification was performed on a Pippin Prep (Sage Science, Beverly, MA, USA) using the 2% agarose gel cassette with ethidium bromide (Sage Science) and size selection range of 300–500 bp. PCR amplification used Q5 Hot Start Master Mix (New England Biolabs, Whitby, ON, Canada) for all libraries. Amplification cycles for all libraries were 98°C for 30 s; *x* cycles of 98°C for 30 s, 65°C for 30 s, 72°C for 30 s; 1 cycle of 72°C for 5 min, where *x* was 18 for all libraries except for SSB, GEO and SUN where *x* was 13. All libraries were sequenced on a HiSeq 2000 (Illumina) as 100 bp paired end sequences with one library per lane. Sequencing was performed at the McGill University and Génome Québec Innovation Centre, Montréal, Canada. Final analysis used a minimum of 12 scallops per population (mean value ± SD of 20.4 ± 2.8 scallops, Table 1).

SNPs were detected using the *de novo* pipeline in STACKS v.0.9999 (Catchen, Amores, Hohenlohe, Cresko, & Postlethwait, 2011). Putative orthologous loci were assembled using *ustacks* with a minimum depth of coverage required to create a stack (m) of five and four maximum nucleotide mismatches (M) allowed between stacks. The catalog of loci was assembled using *cstacks* with a distance allowed between loci in the catalog (*n*) of six. Several other parameter combinations were tested (Table S1a); however, recent work has shown that alternative parameters do not significantly affect population inferences in most cases (Rodríguez‐Ezpeleta et al., 2016), and we elected to use approximate median parameters. Using the *populations* module, only RADtags present in at least 75% of individuals were kept. The final dataset was filtered using PLINK v.1.07 (Purcell, 2009; Purcell et al., 2007) to include SNPs present in at least 75% of individuals with a minor allele frequency greater than 5%. We also tested loci with a MAF of 1%; however the conclusions of our analyses did not change and we saw greater consistency in analyses with a MAF of 5%. Multiple SNPs per RADtag were allowed and treated as separate loci. Furthermore, we excluded individuals with more than 20% missing loci from the analysis. Loci were filtered for Hardy–Weinberg Equilibrium using the program GENEPOP v.4 (Rousset, 2008) and a *p*‐value of .05, excluding loci out of equilibrium in 6 or more populations from the analysis (<0.7% of all loci).

### 2.3 Summary statistics and outliers

We calculated allele frequencies and heterozygosities using the R (R Development Core Team 2012) package *gstudio* (Dyer, 2014) and calculated locus‐specific *F*_ST_ using the program ARLEQUIN v.3.5 (Excoffier & Lischer, 2010). To calculate pairwise linkage disequilibrium [*r* ^2^ (Hill & Robertson, 1968)] between all loci, outlier loci and neutral loci separately using all individuals, we used PLINK v.1.07. Although a variety of methods have been developed to detect loci potentially under selection within a group of populations, individual methods vary in their ability to detect outliers (Narum & Hess, 2011). We used a Bayesian method (Beaumont & Balding, 2004) implemented in the program BAYESCAN v.2.1 (Foll & Gaggiotti, 2008) and an island model implemented in the program ARLEQUIN v.3.5 to determine a candidate list of outlier loci. We ran BAYESCAN with a burn‐in period of 50,000 followed by 100,000 iterations, subsequently identifying outliers in R with a false discovery rate *q*‐value of .05. In ARLEQUIN, we ran 100,000 permutations using 500 demes, 50 groups and a maximum expected heterozygosity of 0.5. *p*‐Values from ARLEQUIN were converted to false discovery rate *q‐*values using *p.adjust* in R, and outliers were determined with a *q‐*value of .05.

### 2.4 Spatial structure

We examined population structure along the range of sea scallops using multiple methods. Hierarchical iterative clustering analysis was conducted using STRUCTURE v.2.2.4 (Pritchard, Stephens, & Donnelly, 2000) through the R package *parallelStructure* (Besnier & Glover, 2013). Results from preliminary BAYESCAN and STRUCTURE analyses guided subsequent analyses (see *3 Results*), after which we analysed nine separate datasets [all loci, neutral loci and outlier loci for each major sample group separately (see *3.3 Observed spatial structure*) (Table S2)] to determine major population groups as well as any minor clusters.

We used Bayesian clustering in STRUCTURE to determine the number of distinct genetic clusters (*K*) present among the 12 sampled populations, running calculations with a burn‐in period of either 50,000 repetitions followed by 200,000 repetitions, or 100,000 repetitions followed by 500,000 repetitions, until algorithm convergence was confirmed. We repeated all runs three times for each *K*, running datasets 1–3 for *K *=* *1–15, datasets 3–6 for *K *=* *1–5 and datasets 7–9 for *K *=* *1–10. To determine the optimal *K* for each dataset, we used the delta *K* method (Evanno, Regnaut, & Goudet, 2005) and processed results using STRUCTURE HARVESTER (Earl & vonHoldt, 2012); runs were grouped and visually displayed using CLUMPAK (Kopelman, Mayzel, Jakobsson, Rosenberg, & Mayrose, 2015). We also completed an analysis of molecular variance (AMOVA) using ARLEQUIN with 25,000 permutations, defining genetic structure following the results from the STRUCTURE analysis. We conducted principal components analysis (PCA) followed by *k*‐means clustering using the R package *adegenet* (Jombart, 2008). This method determines the optimal number of clusters (*k*) in the PCA using the Bayesian information criterion (BIC). The lowest value of the BIC across each value of *k* indicates the number of clusters present in the data. Finally, we constructed neighbour‐joining trees using the programs POPULATIONS (Langella, 1999) and TREEVIEW (Page, 1996) based on estimates of genetic distance among populations (Cavalli‐Sforza and Edwards chord distance, *D*_*c*_) with 1000 bootstrap replications on individuals. Following the results from the spatial structure analyses (see *3 Results*), we also completed preliminary hybrid detection using NEWHYBRIDS (full details in *Supporting Information*).

### 2.5 Estimates of dispersal and connectivity

We explored two approaches to estimate average per generation dispersal distance, both of which make different assumptions regarding the underlying model of gene flow. First, we used an IBD model which assumes a linear one‐dimensional stepping stone for gene flow [see Bradbury and Bentzen (2007)]. This approach used linear regression between pairwise population *F*_ST_/(1−*F*_ST_) and spatial distances based on two measures of geographic distance: approximate ocean distances following prevailing currents estimated in GOOGLE EARTH (Google 2013) using average current patterns in the Northwest Atlantic and the shortest ocean‐based distance (within 5 km of the shoreline) calculated using the R package *marmap* (Pante & Simon‐Bouhet, 2013), where distance was calculated excluding positive elevation (land). We calculated IBD separately using all loci, outlier loci and neutral loci and for all sampled populations and each major sample group separately (see *3.3 Observed spatial structure*). We performed Mantel tests to ascertain the significance of every IBD relationship. Adult–offspring dispersal distance estimates were calculated following Rousset (1997) using the slope of the IBD relationship. We estimated adult density values required for the IBD methods from DuPaul and Rudders (2008); Mason, Sameoto, and Metaxas (2014); and Kelly (2007) for several areas within the study range and used them as density proxies along the entire species range. Furthermore, because census estimates of density likely differ from effective density, we explored the sensitivity of the dispersal estimate to a range of density values several orders of magnitude above and below the actual estimates used.

The second approach employed a clinal model of gene flow following Barton and Gale (1993); Lenormand, Guillemaud, Bourguet, and Raymond (1998); and Sotka and Palumbi (2006). Here, clines in allele frequency for outlier loci and a random subset of 500 neutral loci were estimated using the R package *HZAR* (Derryberry, Derryberry, Maley, & Brumfield, 2014) using 100,000 iterations following a 10,000 iteration burn‐in period. We used population‐specific allele frequencies for all loci tested and estimated distances from the northernmost population (SUN) along a one‐dimensional transect that included all populations using GOOGLE EARTH (Google Inc, 2013). Four cline models and a null model were generated for each locus, and cline model selection used AICc criteria followed by a log‐likelihood cut‐off of −10. Models tested included fixed or free minimum and maximum allele frequency values and either no exponential cline tails or tails at both ends of the cline. We determined cline width from the best fit model and used cline width in estimates of adult–offspring dispersal distance. Here, adult–offspring dispersal distance estimates followed Sotka and Palumbi (2006) using cline width and linkage disequilibrium (average locus‐specific *r* ^2^, see *2.3 Summary Statistics and Outliers*) to determine the standard deviation in parent–offspring distance. Differences between cline width and dispersal estimates in neutral and outlier loci were assessed using the Welch two‐sample *t*‐test.

## 3 Results

### 3.1 RADseq

Following initial filtering, we retained 19672 RADtags (14.9% of initial RADtags) present in more than 75% of individuals. Read count / individual / RADtag ranged from 5 to 1209 (average 56.12 ± 46.64 reads / individual / RADtag) (Figure S1). Further filtering (SNPs present in 95% of individuals, individuals with <20% missing data, MAF >5%) reduced our dataset to 245 individual scallop samples (97.2% of sequenced individuals) and 7216 SNPs (4.2% of Initial SNPs) (Table 2). Applying alternative parameters sets produced similar SNP numbers (Table S1b), and we found that altering parameters did not affect our conclusions. The 7163 SNPs in HWE that met all quality control standards were used in all subsequent analyses.

**Table 2:** Number of *P. magellanicus* individuals and number of SNP loci included in initial sequencing and final analysis following quality control (QC)

Parameter	Value
Individuals sequenced	252
*Individuals following QC*	245 (97.2% of Individuals sequenced)
Initial RAD tags	131897
*RAD tags following QC*	19672 (14.9% of Initial RAD tags)
Initial SNPs	173482
*SNPs following QC*	7216 (4.2% of Initial SNPs)
SNPs in HWE	7163 (99.3% of SNPs following QC)
*Outlier SNPs*	112 (1.6% of SNPS in HWE)
*Neutral SNPs*	7051 (98.4% of SNPs in HWE)

### 3.2 Summary statistics, differentiation and linkage

**Figure 2:**
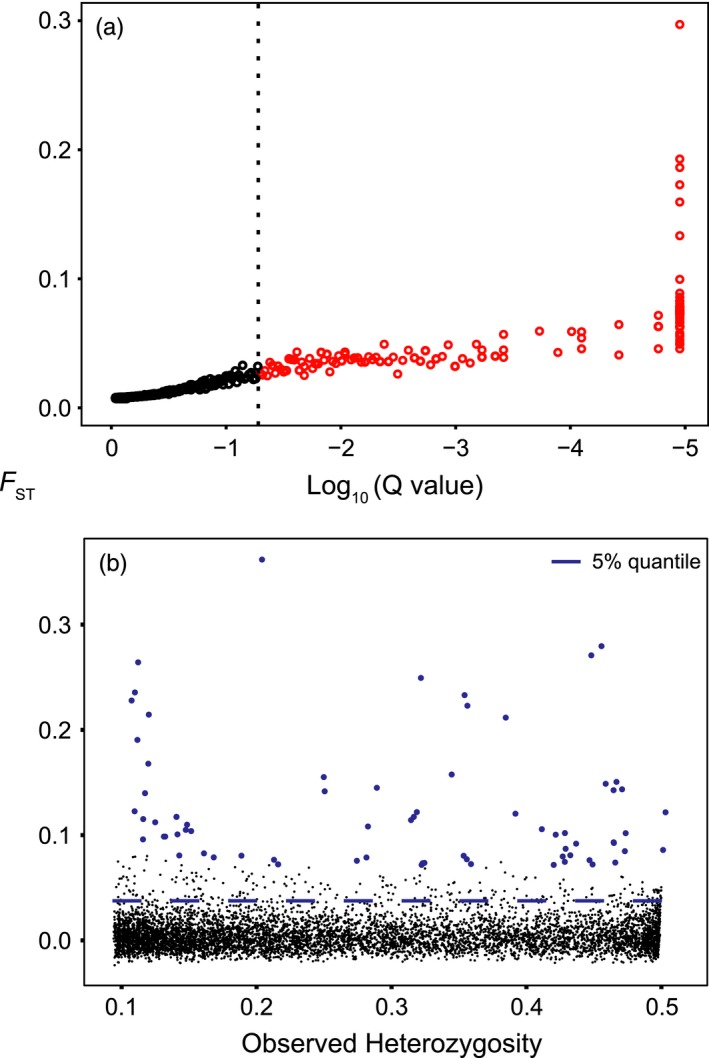
Results from (a) the Bayesian test for selection completed using the program BayeScan and (b) the hierarchical island model test for selection completed using the program Arlequin for 7163 loci sequenced in 12 populations of *P. magellanicus*. BayeScan outliers are defined as all loci with a *q*‐value higher than .05 (highlighted in red). Arlequin outliers are defined as the loci that fall above the simulated 5% quantile of *F*_ST_ versus Heterozygosity (*q *≤ .05, highlighted in blue)

For the final dataset, minor allele frequency (MAF) averaged 0.1855 ± 0.1253 (mean ± SD), expected heterozygosity averaged 0.2710 ± 0.1333 (mean ± SD) and locus‐specific *F*_ST_ averaged 0.0066 ± 0.0198 (mean ± SD) (full histograms presented in Figure S2). Of the final 7163 SNPs, 112 SNPs (1.6%) were identified as outliers by BAYESCAN, leaving 7051 (98.4%) in the neutral dataset. The outlier lists from BAYESCAN and ARLEQUIN were similar, with only four of the 72 loci detected using ARLEQUIN missing from the BAYESCAN list (Figure 2, Table S3). Because the outlier lists were very similar, and to ensure we captured as much potential outlier variation as possible in our analyses, we focused on the BAYESCAN list for all subsequent analysis of outlier loci. Pairwise population‐specific *F*_ST_ calculated using ARLEQUIN was higher for outlier loci than either neutral loci or all loci (Table S4), with average values of 0.005 ± 0.006 (mean ± SD) using all loci, 0.003 ± 0.005 (mean ± SD) using neutral loci and 0.094 ± 0.070 (mean ± SD) using outlier loci. In all cases, pairs of populations containing one north and one south population (see *3.3 Observed spatial structure*) yielded maximum values with the highest differentiation (average north/south pairwise *F*_ST_ was 0.008 ± 0.004 (mean ± SD) using all loci, 0.004 ± 0.004 (mean ± SD) using neutral loci and 0.15 ± 0.04 (mean ± SD) using outlier loci).

Average pairwise *r* ^2^ values indicating linkage disequilibrium were higher in outlier loci than neutral loci but even the outlier values remained low overall (outlier loci: 0.0258 ± 0.0829, neutral loci: 0.0044 ± 0.0098, all loci: 0.0044 ± 0.0098, mean ± SD). Within the outlier loci, a few small pockets of higher linkage seemed to drive the higher average *r* ^2^ value (Figure S3), likely because several outlier SNPs were from the same RADtag. Overall, 11 RADtags were represented multiple times in the outlier loci (27 loci total, between 2 and 4 SNPs per RADtag). However, because overall *r* ^2^ values were low, we did not filter loci because of linkage disequilibrium in subsequent analysis.

### 3.3 Observed spatial structure

**Figure 3:**
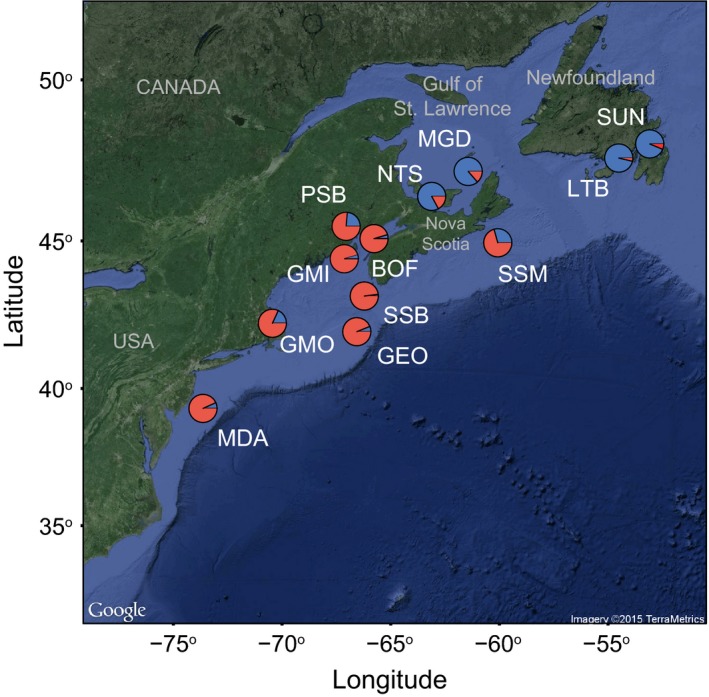
Map of the proportion of each of the 12 *P. magellanicus* populations assigned to two population groups (blue and red) identified in the program STRUCTURE using outlier loci and the ΔK method to select the optimal number of genetic clusters in the data

**Figure 4:**
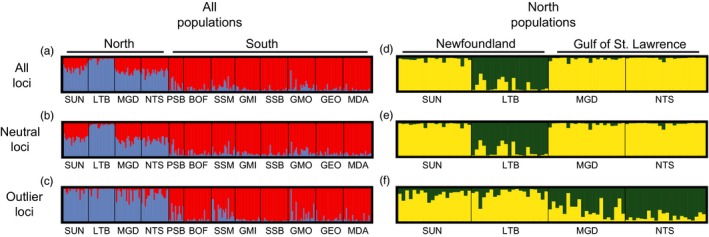
Plots of individual admixture for 12 populations of *P. magellanicus* at *K *=* *2 determined using the program STRUCTURE and the ΔK method to select the optimal number of genetic clusters in the data. Results are presented for all populations at (a) all loci, (b) neutral loci and (c) outlier loci as well as four north populations at (d) all loci, (e) neutral loci and (f) outlier loci

Different methods of determining population structure generally produced similar results (Table S5). Bayesian population structure analysis in STRUCTURE clearly splits north and south groups using three datasets; *K *=* *2 was best supported for all loci, neutral loci and outlier loci (Figure S4). The north group consisted of four samples from Newfoundland and the Gulf of St. Lawrence, whereas the south group contained the remaining eight samples from south of the Scotian Shelf (Figures 3 and 4a, b, c). Further hierarchical structure analysis on the north group revealed a split into two sample groups, however, the pattern of structure differed among the outlier and neutral loci (*K *=* *2 in all cases, Figure S5). When using all loci and only neutral loci, LTB clustered separately from all other north populations (Figure 4d, e). When using outlier loci, LTB and the SUN sample clustered together separate from the Gulf of St. Lawrence samples (MGD and NTS) (Figure 4f). Structure analysis of the eight south populations revealed no clear clustering or evidence of differences among samples (Figure 4a, b, c). AMOVA to explore the amount of variation explained by this subdivision showed that the split between north and south sample groups explained a small percentage of total genetic variance in all loci and neutral loci (all loci = 0.58%, neutral loci 0.40%). However, AMOVA results for outlier loci differed from the other datasets with 11.0% of all variation explained by the split between north and south sample groups (Table 3).

**Table 3:** Analysis of molecular variance (AMOVA) among 12 populations of *P. magellanicus*, among regional groups of populations identified by Structure analysis, and among individuals within populations using (A) all loci, (B) neutral loci, and (C) outlier loci

Source of variation	*df*	Proportion of variation	*p*‐value
(A)			
Among groups	1	0.58	<.001
Among populations within groups	10	0.09	<.001
Among individuals within populations	233	5.43	<.001
Within individuals	245	93.90	<.001
(B)			
Among groups	1	0.40	<.001
Among populations within groups	10	0.02	<.001
Among individuals within populations	233	5.50	<.001
Within individuals	245	94.08	<.001
(C)			
Among groups	1	11.00	<.001
Among populations within groups	10	4.24	<.001
Among individuals within populations	233	1.71	<.001
Within individuals	245	83.06	<.001

**Figure 5:**
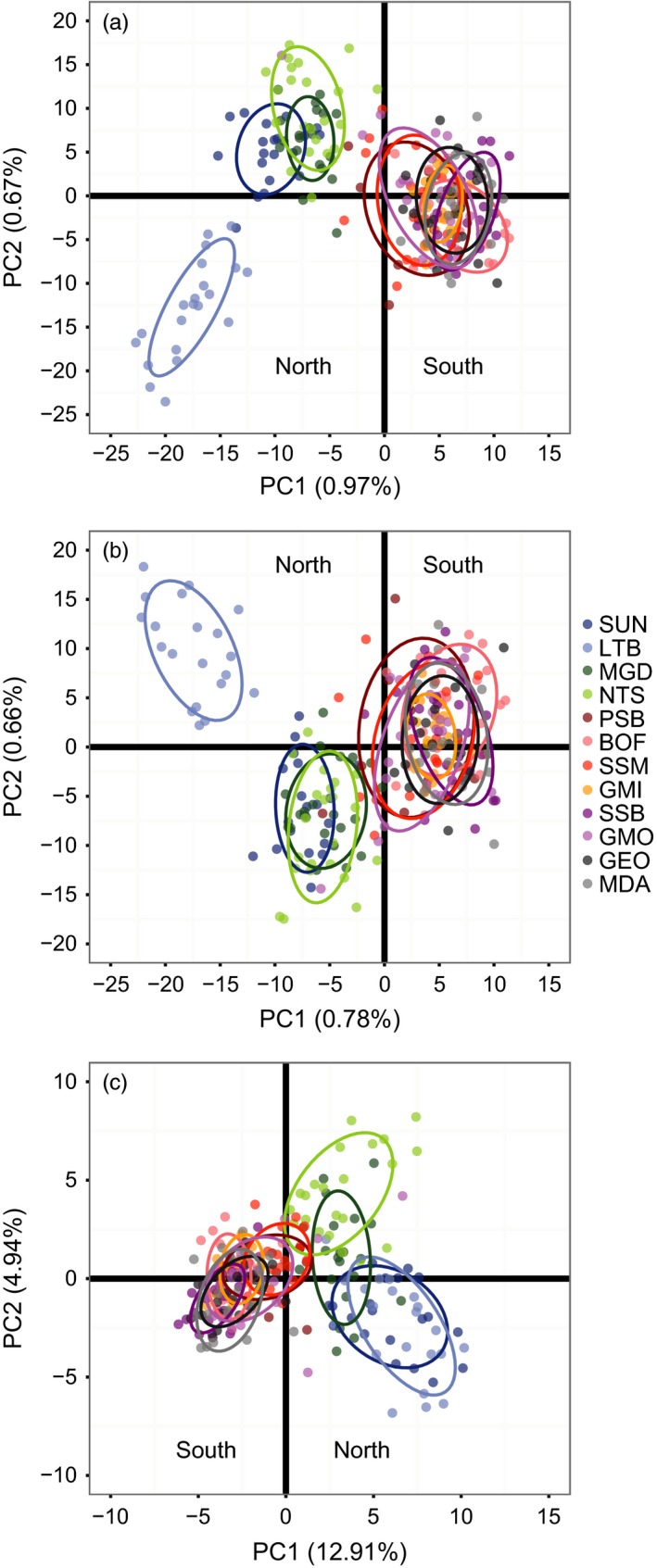
Principal components analysis plots for (a) all loci, (b) neutral loci and (c) outlier loci in 12 populations of *P. magellanicus*

**Figure 6:**
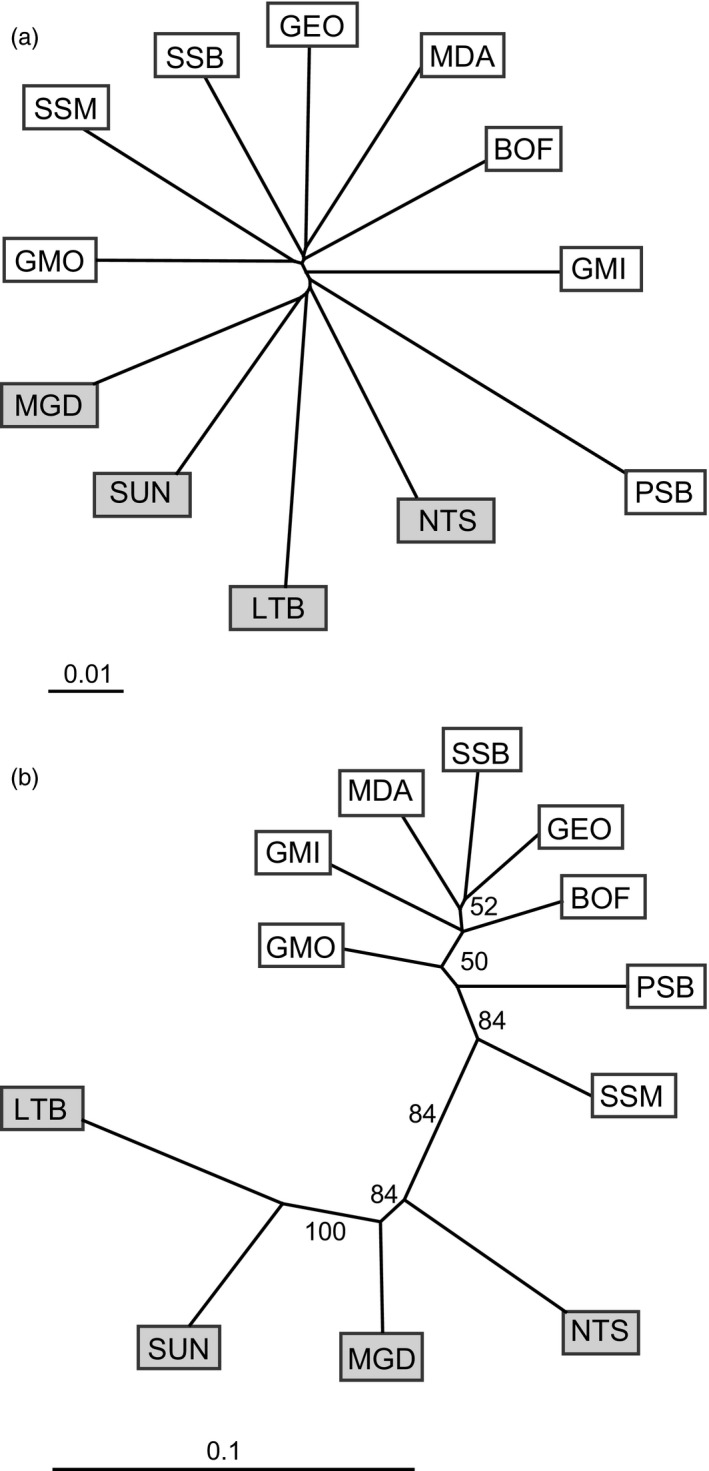
Neighbour‐joining trees for Cavalli–Sforza and Edwards chord distance (*D*_*c*_) between 12 populations of *P. magellanicus* for (a) neutral loci and (b) outlier loci. North populations are highlighted in grey, south populations in white and bootstrap values greater than 50% are shown

In addition to the STRUCTURE analysis, we used principal components analysis (PCA) and neighbour‐joining trees (NJ) to explore spatial relationships in two dimensions. PCA on all sets of loci split north and south samples along the first principal component, similar to the division in the STRUCTURE analysis (Figure 5). This first principal component (PC) explained 0.97%, 0.78% and 12.91% of the total variance explained by the analysis using all loci, neutral loci, and outlier loci, respectively (Figure S6). PCA using all loci and neutral loci further separated LTB from the other north populations along the second principal component, but this pattern was not seen in the outlier loci (comparable to the STRUCTURE results). *K*‐means clustering only identified one genetic group when using all loci and the neutral loci (*k *=* *1), but with the outlier loci detected the same north–south split seen in the STRUCTURE results as well as further structuring within the regional sample groups (*k *=* *4, Figure S7, Figure S8). Neighbour‐joining trees showed the same north–south split seen in other analysis; however, only the outlier loci supported the split (Figure 6) as indicated by a bootstrap support value of 84%. Hybrid detection identified several populations with North/South hybrid individuals, most notably in the middle of the geographic sampling range near the discontinuity between northern and southern clusters (see *Supporting Information* for full results).

### 3.4 Estimates of dispersal and connectivity

**Figure 7:**
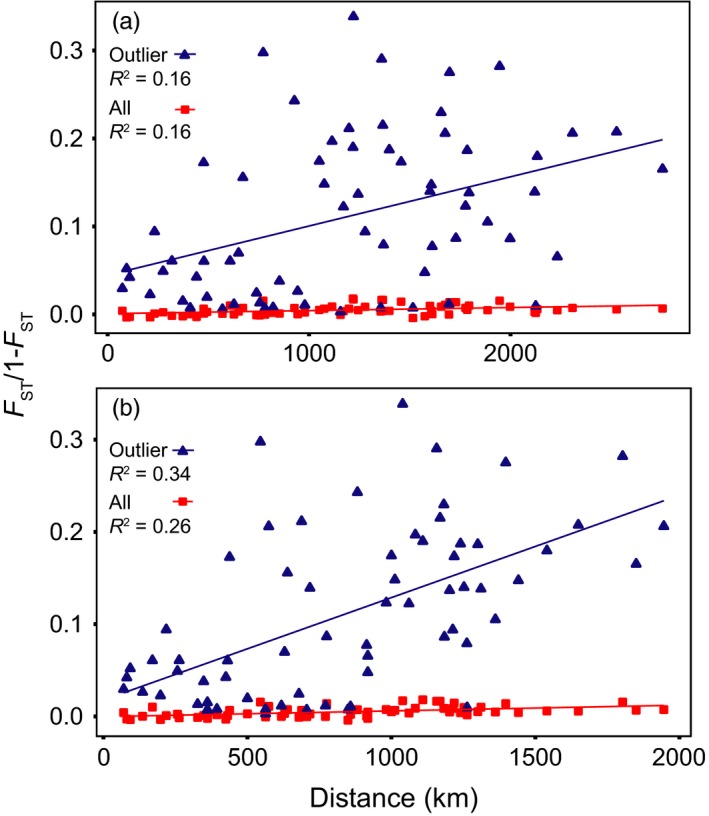
Isolation by distance plot of *F*_ST_/1−*F*_ST_ versus population pairwise distance for 12 populations of *P. magellanicus* using (a) approximate current‐based distance (all loci: *p* < .05, outlier loci: *p* < .01) and (b) shortest ocean‐based distance (all loci: *p* < .05, outlier loci: *p* < .001) for all loci (red squares) and outlier loci (blue triangles)

We examined IBD relationships using 18 different combinations of samples, loci and population distance measures. These included using all populations, north populations or south populations; using all loci, neutral loci or outlier loci; and using current‐based pairwise population distance or the shortest ocean‐based distance. Only five of these 18 IBD relationships were significant. Using the approximate ocean current‐based pairwise geographic distance, we found a significant IBD relationship only when using all 12 populations and all loci (*R* ^2 ^= .1575, *p* < .05) or the outlier loci (*R* ^2 ^= .1648, *p* < .01) (Figure 7a). When using the shortest ocean‐based distance, we found a significant IBD relationship when using all 12 populations and all loci (*R* ^2 ^= .2609, *p* < .001) or the outlier loci (*R* ^2^ = .3363, *p* < .001) (Figure 7b). Using the shortest ocean‐based distance and only the north populations, we found a significant IBD relationship using the outlier loci (*R* ^2 ^= .6309, *p* < .05). However, as the spatial analysis above clearly indicated the presence of two dominant clusters driving the IBD relationship, this pattern was not consistent with a one‐dimensional stepping stone framework assumed by the Malécot's lattice model (Malécot, 1955) and the approach outlined by Rousset (1997) for estimating dispersal distance. As a result, dispersal estimates from IBD analysis were consistently unrealistically small (<15 km, Table S7) and not considered further.

**Figure 8:**
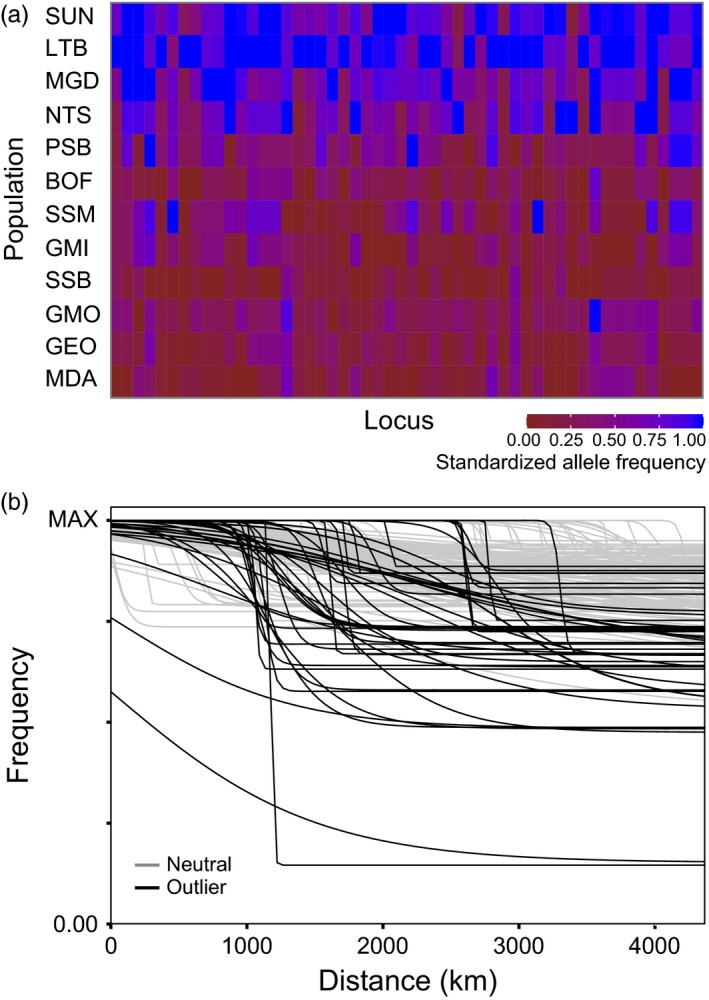
(a) Heat map of population‐specific standardized allele frequencies for 48 clinal outlier loci in 12 populations of *P. magellanicus*. (b) Plot of clines in allele frequency in 12 populations of *P. magellanicus* as a function of the distance in kilometres from the furthest north population (SUN) for clinal neutral loci (*n* = 123, 24.6% of tested loci, grey) and clinal outlier loci (*n* = 48, 42.9% of tested loci, black)

We also used evidence of clinal trends in allele frequency to estimate average per generation dispersal distance. Of the 112 outlier loci tested, 48 (42.9%) showed significant clines (non‐null model and log‐likelihood >−10) (Figure 8a). Of the 48 clinal loci, 16 (33.3%) had fixed scaling and no exponential tails in allele frequencies and the remaining 32 (66.7%) had free scaling and no exponential tails. Average outlier cline width was 1157.0 ± 1268.6 (mean ± SD) km, with a minimum cline width of 14.4 km and a maximum cline width of 4524.1 km. We next examined a randomly selected subset of 500 neutral loci for clinal patterns (7.09% of total neutral loci). Of these 500, 377 (75.4%) showed no cline in allele frequencies. Of the 123 loci showing clinal patterns, 68 (55.3%) had fixed scaling and no exponential tails, and 55 (44.7%) had free scaling and no exponential tails. Within these 123 loci (24.6% of the 500 tested), average cline width exceeded the outlier loci, at 2523.8 ± 2144.1 km (mean ± SD), with a minimum cline width of 3.4 km and a maximum cline width of 4529.8 km. Cline widths differed significantly between outlier and neutral loci (*p* < .001) (Figure 8b).

The estimated standard deviation of parent–offspring distance when using clines from outlier loci was 373.1 ± 407.0 km (mean ± SD). The estimated standard deviation of parent–offspring distance when using clines from neutral loci was higher than the outlier loci estimate, at 641.0 ± 544.6 km (mean ± SD). Both estimates are lower than the average shortest ocean‐based pairwise distance between our sample sites (840.4 ± 464.5 km, mean ± SD) and differ significantly from one another (*p* = .0007).

## 4 Discussion

Successful management and conservation of exploited and threatened species require an accurate understanding of population connectivity and dispersal patterns (Allendorf et al., 2010). In marine species, estimates of dispersal and connectivity remain rare largely because of the difficulty in tracking small pelagic larval stages to settlement (Bradbury, Laurel, Snelgrove, Bentzen, & Campana, 2008). Here, we used RADseq‐derived SNPs to explore spatial patterns of connectivity and estimate dispersal in a commercially exploited marine bivalve, *P. magellanicus*. Our results show significant population differentiation and structure across the range of *P. magellanicus* despite high dispersal potential during a pelagic larval stage. Our estimates of dispersal indicate geographically restricted connectivity, particularly when using outlier loci suggesting a role for selection in determining realized connectivity and limiting gene flow. Accurate knowledge of sources of larvae and dispersal patterns such as those revealed here can significantly influence population persistence into the future (Hastings & Botsford, 2006); management strategies that incorporate results from studies mapping population structure and dispersal patterns may be among the most effective (Fogarty & Botsford, 2007) and the use of genomic tools such as those used here can directly facilitate successful conservation and fisheries management (e.g. Miller, Mundy, & Mayfield, 2014; da Silva, Appleyard, & Upston, 2015).

### 4.1 RADseq and marine connectivity

The use of RADseq in marine species has provided unprecedented access to measures of genomewide variation with obvious applications for marine management and conservation. SNPs generated using RADseq techniques have been used for a variety of research aims, from identifying historical phylogeography to resolving contemporary population structure (Chu, Kaluziak, Trussell, & Vollmer, 2014; Combosch & Vollmer, 2015; Corander, Majander, Cheng, & Merila, 2013; Ogden et al., 2013). Our results (i.e. number of SNPs and outliers) are consistent with previous work using RADseq, providing further support for the view that RADseq‐based genome scans can generate 1000s of SNPs in nonmodel marine species with direct application to management and conservation needs. The number of loci from these studies is one to several orders of magnitude larger than the number of loci used in studies utilizing other genetic markers. In *P. magellanicus*, for example, two previous population genetic studies both used less than 10% of the loci used in our study [six microsatellites in Kenchington et al. (2006), 634 AFLPs in Owen and Rawson (2013)]. The sheer number of markers generated using RADseq and their placement across the entire genome of an organism are predicted to increase accuracy and power of statistical tests of differentiation and spatial patterns (Allendorf et al., 2010; Waples, 1998).

### 4.2 Detection and influences of selection

The ability to detect loci potentially under directional selection offers a significant advantage to RADseq‐based genome scans over traditional approaches in marine population genetic studies (Gagnaire et al., 2015). Identifying markers potentially under selection can improve the accuracy of conclusions drawn from population genetic studies; failing to account for the effects of selection could lead to overestimation of neutral differentiation and underestimation of gene flow. Using outlier loci may also increase spatial resolution (Hellberg, 2009), providing opportunities to track individuals and predict adaptive differences (e.g. Therkildsen et al., 2013). Separating outlier and neutral loci allows us to disentangle the effects of selection and underlying neutral variation (and gene flow) within sea scallops, generating a more complete picture of population connectivity in this species. Undoubtedly, each approach includes some identification error, but the combination of several outlier detection methods can help reduce rates of false positives (Gagnaire et al., 2015). Our list of outlier loci was largely robust to the assumptions of differing approaches because there was substantial overlap in the loci identified by both BAYESCAN and ARLEQUIN, supporting the outlier status of these loci. The number of outlier loci detected in our study (112, 1.6% of all loci examined) compares favourably with numbers of outlier loci detected and used in other studies of marine organisms that examine intraspecific variation (Guo, DeFaveri, Sotelo, Nair, and Merila (2015): 0.99% of identified SNPs; Milano et al. (2014): 4.59%; Hess, Campbell, Close, Docker, and Narum (2013): 3.65%; Bradbury et al. (2013): 5.2%; De Wit and Palumbi (2013): 3.2%; and Bourret et al. (2013): 2.6%). Although low, this number nonetheless remains reasonably consistent with studies suggesting approximately 5%–10% of a genome in marine species show signatures of selection (Nosil, Funk, & Ortiz‐Barrientos, 2009; Strasburg et al., 2012).

### 4.3 Spatial population structure

Many RADseq studies of marine species report fine‐scale geographic structure (Benestan et al., 2015; Catchen et al., 2013; Reitzel et al., 2013). We observed significant population structure along the range of *P. magellanicus* separating sampling locations into two distinct groups – north and south of Nova Scotia, Canada. These results mirror population structure detected in other marine species in the Northwest Atlantic, including *Homarus americanus* (Benestan et al., 2015) and *G. morhua* (Bradbury et al., 2010, 2013, 2014), and build on smaller‐scale levels of differentiation reported among scallop populations (Kenchington et al., 2006; Owen & Rawson, 2013). The presence of two distinct groups raises the possibility of dispersal and subsequent hybridization. Three of the north populations (SUN, MGD and NTS) show evidence of admixture when examined using neutral loci and all loci together, indicating some gene flow between the north and south groups. The highest frequency of hybrids occurs in populations within the centre of our sampling locations (near the centre of the genomic cline), particularly the offshore sites.

Several causes may have contributed to the genetic separation of northern and southern populations. First, historical demographic processes could play a role through vicariance followed by secondary contact. Previous studies have evaluated hypotheses of secondary contact in the region for other species [including Atlantic cod (Carr & Marshall, 2008) and several wolffish species (McCusker & Bentzen, 2010)], but reported no evidence of secondary contact; the authors' analyses identify postglacial expansion as the most likely demographic scenario. Further study is likely needed to determine the demographic history of sea scallops in the Northwest Atlantic and the role that historical vicariance might have played in explaining the observed genetic clines. Second, for marine species with planktonic larval stages, larval dispersal is expected to contribute significantly to spatial population structuring (Bradbury & Snelgrove, 2001; Bradbury, Laurel, Snelgrove, et al., 2008) although patterns of resultant connectivity can be complex. Marine larvae are unlikely to occur uniformly through the water column (Manuel, Burbridge, Kenchington, Ball, & Odor, 1996a; Tremblay & Sinclair, 1990a,b), and variation in currents could influence dispersal distances, directions, survival and the source populations of dispersing larvae (Kordos & Burton, 1993; Metaxas, 2001; Tilburg, McCartney, & Yund, 2012; Townsend, Thomas, Mayer, Thomas, & Quinlan, 2006). Finally, variation in temperature and other environmental factors between populations may contribute to local adaptation, further influencing the differential survival of dispersing and recently settled larvae and contributing to genetic population structure.

Our cline analysis showed evidence that multiple processes contribute to the observed patterns of population structure. Although outlier loci might be expected to display narrower clines than neutral loci, our results show clinal patterns in some of our neutral loci with a smaller minimum cline width in neutral loci than outlier loci. The presence of a clinal pattern in allele frequencies is not itself indicative of selection (Vasemägi, 2006), and despite the smaller minimum cline width in neutral loci, we found a smaller and less variable average cline width in outlier loci than neutral loci. Furthermore, we found larger cline height (change in allele frequencies) in outlier loci. Considering the strength of the genetic break in outlier loci and the larger change in allele frequencies in clinal outlier loci compared to neutral loci, our results may be indicative of adaptation to regional environmental conditions. Further research into potential drivers of population structure in the sea scallop might help determine how selective forces may be influencing population dynamics in the Northwest Atlantic.

Not surprisingly, all of our analyses found stronger genetic differentiation and population structure signals in outlier loci than when examining neutral loci alone or all loci together. This finding is consistent with both other studies that detected higher levels of structure and increased spatial resolution of population structure with outlier loci [(Bradbury et al., 2010; Hemmer‐Hansen, Therkildsen, Meldrup, & Nielsen, 2014; Milano et al., 2014), but see Moore et al. (2014) for an exception] and the hypothesis of genomic islands of adaptive divergence (Nosil et al., 2009). In the northern population cluster, both strong isolation of Little Bay (LTB) (leading to genetic drift) and a semi‐permeable barrier to gene flow (allowing neutral loci to spread between locally adapted groups) could cause the patterns we see in neutral loci that are not evident in outlier loci (Barton & Bengtsson, 1986; Barton & Hewitt, 1985; Feder & Nosil, 2010; Harrison & Larson, 2014; Nosil et al., 2009). Comparing tails of introgression around a genetic cline can help determine the likely cause of the differences in population structure patterns observed between outlier and neutral loci (Gagnaire et al., 2015). We find little evidence of introgression in our neutral cline plots, suggesting that the admixture patterns seen among northern populations result from strong isolation of LTB away from all other populations rather than a semi‐permeable barrier to gene flow. Circulation patterns near LTB may retain larvae from local populations and limit larval dispersal out of Placentia Bay (Bradbury, Snelgrove, & Fraser, 2000; Bradbury, Laurel, Robichaud, et al., 2008), preventing dispersal to other populations and reflecting local larval retention and increased genetic drift. Ultimately, differences in spatial patterns between putative outliers and neutral markers (in the north cluster in particular) may reflect influences of differing structuring forces such as selection and drift but attributing spatial variation to these factors will require additional genetic data resources and study.

### 4.4 Estimates of dispersal

Estimating dispersal distance in marine species remains a significant challenge (Selkoe & Toonen, 2011). Standard methods of estimating dispersal include (but are not limited to) drifter studies and biological–physical modelling, PLD, chemical tracking, direct observation, assignment tests and use of natural or artificial markers (Bradbury, Laurel, Snelgrove, et al., 2008; Cowen & Sponaugle, 2009; Hedgecock et al., 2007; Levin, 2006; Saenz‐Agudelo, Jones, Thorrold, & Planes, 2009; Selkoe & Toonen, 2011; Thorrold, Zacherl, & Levin, 2007; Thorrold et al., 2002). Our estimates of effective dispersal here (ranging from approximately 300 to 600 km per generation) compare directly with estimates for other marine invertebrates and fish in eastern North America (Bradbury, Laurel, Robichaud, et al., 2008; Kinlan & Gaines, 2003) and, particularly, *P. magellanicus* on George's Bank and the Mid‐Atlantic Bight where modelling studies of scallop larvae identified some local retention and dispersal between adjacent populations (Davies, Gentleman, DiBacco, & Johnson, 2014, 2015; Gilbert et al., 2010; Tian et al., 2009a,b). Given the long planktonic larval period in scallops, some correlation between population structure and the direction and nature of coastal circulation patterns may be expected. Previous work in sea scallops associated genetic structure with the dominant ocean currents, supporting larval dispersal as the main structuring agent (Kenchington et al., 2006); however, patterns of larval movement differed based on the depth of model particles. Given larval *P. magellanicus* have been previously shown to exhibit diel behaviour (Tremblay & Sinclair, 1990a), the assumption that surface currents approximate dispersal potential might be tenuous. Our observation that the shortest ocean‐based distance predicted genetic spatial structure better than current‐based geographic distance suggests our approximations of circulation may not capture the complexity of larval dispersal. This observation may also reflect the influence of variation in postsettlement processes (e.g. mortality) (Bradbury, Campana, & Bentzen, 2008a; Clarke, Munch, Thorrold, & Conover, 2010) associated with climatic variation expected across this range (Townsend et al., 2006) on the realized connectivity of the system.

Genetic methods of estimating and inferring dispersal patterns reflect effective dispersal (i.e. the subsequent survival and reproduction of dispersers) rather than strict movement among populations. Comparison of dispersal estimates based on neutral and outlier loci may allow some inference of the roles that dispersal and selection play in regulating connectivity. Our observation that the average estimates of dispersal based on the outlier loci were smaller than those based on neutral loci supports a hypothesis that selection and differential survival may be important in limiting effective dispersal and connectivity in sea scallops. Other studies report similar observations for coastal fish species elsewhere, detecting genetic structure at smaller geographic scales than dispersal would suggest (Bradbury, Campana, & Bentzen, 2008b; Clarke et al., 2010). As may be expected, both of the methods used to estimate dispersal in our system make inherent assumptions. Previous work demonstrates that IBD itself is robust to deviations from some model assumptions (Leblois, Estoup, & Rousset, 2003; Leblois, Rousset, & Estoup, 2004) with clear successes in estimating local dispersal (Broquet, Ray, Petit, Fryxell, & Burel, 2006; Rousset, 1997; Sumner, Rousset, Estoup, & Moritz, 2001). However, the use of IBD to estimate dispersal distance in sea scallops may be inappropriate for the patterns of population structure detected in the system. A strong cline between north and south populations characterizes the primary population structure observed in sea scallops. This pattern does not follow the classic stepping stone model used in IBD and likely biases the analysis. The clinal method therefore seems most applicable and although limitations include errors in LD calculations, equations that assume selection/dispersal balance and violation of assumptions by long‐distance dispersal (Sotka & Palumbi, 2006), the potential estimation errors resulting from these assumptions may be minor in our system. A steep cline primarily drives the structure we identified within scallops in contrast to the patterns assumed in the gradual island or stepping stone model used in IBD analyses. In our case, cline‐based estimates likely produce more accurate dispersal values and our results reflect this improved accuracy. The cline‐based estimates are smaller than the average pairwise distance found between our populations indicating that limited dispersal may add significantly to population structure within the sea scallop.

### 4.5 Implications for management

The results from this study can directly inform sea scallop fishery management in both Canada and the USA. Sustainable harvesting of sea scallops depends on harvest levels and the degree of connectivity among areas of suitable habitat. Management strategies to limit harvesting are implemented regionally and locally and include catch quotas, gear limitations, seasonal restrictions and the implementation of fisheries closed areas (e.g. Kelly, 2007). Spatial management and conservation measures can integrate the population boundaries identified here. The clear separation between northern and southern population clusters in the sea scallop, particularly in outlier loci, can be valuable for diagnosing stocks even when adaptive significance is unknown (Russello, Kirk, Frazer, & Askey, 2012; Shafer et al., 2015). By comparing results from outlier and neutral loci (Funk, McKay, Hohenlohe, & Allendorf, 2012), we identified the potential isolation of Placentia Bay in NL (LTB) from all other northern samples. This potential structuring warrants further consideration both from an adaptive genomics and a management perspective.

### 4.6 Limitations

Although genetic methods, and RADseq in particular, offer great potential for measuring marine connectivity (Gagnaire et al., 2015), several limitations and caveats are worth considering. The use of nonrandom missing data may affect population genetic inferences and conclusions based on RADseq data (Arnold, Corbett‐Detig, Hartl, & Bomblies, 2013; Gautier et al., 2013). However, Arnold et al. (2013) found *F*_ST_ to be relatively robust to missing data compared to other differentiation estimates. Arnold et al. (2013) also recommend complete trimming of loci with missing data; we trimmed our loci to maximum 20% missing data; however, loci with missing data comprised a very small proportion of our total loci and likely had no substantial influence on our results. As discussed previously, local adaptation and selection among different populations may influence the conclusions of population genetic studies by leading to overestimation of the differentiation between populations, promoting inaccurate estimates of migration and gene flow between populations if the possible influence of selection is not considered. We separated loci for analysis both to identify potential regions of local adaptation but also to generate a more conservative and potentially accurate pattern of dispersal and connectivity among sea scallop populations. Genetic methods characterize effective connectivity, or only the contributions of dispersers that survive and reproduce, and may miss instances of larval movement without subsequent reproduction within the new population. For marine management, however, the effective movement and survival of dispersers and the contribution of dispersers to population stability generally represents the most important measure. Despite somewhat limited availability of samples, we included scallops spanning a wide range of ages wherever possible to avoid conclusions based on the genetic signatures of rare events (e.g. unusual currents or storms facilitating abnormal dispersal). In addition, repeated sampling, more individuals per population and more detailed age structure analyses could help confirm the stability of patterns of population structure over time. Finally, accurate dispersal estimates using the methods employed in our analysis require an accurate understanding of cline shape. The distribution of our sampling sites limits our ability to accurately detect cline shape within the transition zone between our north and south population clusters. Further sampling will provide more detailed information regarding the cline shape and allow future researchers to more accurately describe area of population cluster separation.

## 5 Summary

Using RADseq‐derived SNPs, we describe range‐level population structure in sea scallops, building on work that detected smaller‐scale differentiation using microsatellites (Kenchington et al., 2006) and AFLPs (Owen & Rawson, 2013). Significant isolation between the northern and southern regions of the species distribution mirrored patterns in other Northwest Atlantic species. Estimates of dispersal using genomic clines, likely the most appropriate approach for our system, indicate moderate potential dispersal within sea scallops. However, variables other than larval transport may also drive population structure, including demographic processes and adaptation. Our comparison of these methods highlights the fact that assumptions involved in these different methods of estimating dispersal means some may not be appropriate in all cases. Patterns in population structure differed when using neutral and outlier loci indicating that selection and local adaptation may play a role in sea scallop population dynamics. The major population structure identified, as well as the potential for adaptation, offers valuable information for management of this economically important species. The same factors that structure sea scallop populations presumably affect other species in the region with similar life histories, and comparison of these species with associated environmental and oceanographic variation in the area may provide significant insights into prevalent factors influencing regional population differentiation and adaptation.

## Data Accessibility

All raw sequences are available at NCBI SRA Bioproject # PRJNA340326, Biosample #s SAMN05712457 ‐ SAMN05712468. The 7216 SNPs selected before HWE filtering (VCF format) and geographic distance matrices used in this study have been deposited in Dryad at http://dx.doi.org/10.5061/dryad.2nh23. All corresponding sample names, codes and locations are in Table 1 and Figure 1.
