# Supplementary material for: Identifying patterns of dispersal, connectivity and selection in the sea scallop, Placopecten magellanicus, using RADseq‐derived SNPs
Source: Evol Appl. 2016 Nov 2;10(1):102–17. doi: 10.1111/eva.12432 (PMC5192885; doi:10.1111/eva.12432)
Supplement: Supplementary file 1 [file EVA-10-102-s001.docx]

**Supporting Information**

for

**Identifying patterns of dispersal, connectivity, and selection in the sea scallop, *Placopecten magellanicus,* using RAD-seq derived SNPs**

**Methods**

*Hybrid detection*

We implemented the program NEWHYBRIDS (Anderson and Thompson 2002) to detect potential hybrids between the northern and southern population clusters (see *Results*). We used the R package *hybriddetective* (Wringe et al. in prep) to create three simulated data sets of pure northern, pure southern, F1, F2, and backcrossed hybrid individuals based on a random proportion of 0.9 of 50 individuals considered as pure from both the northern and southern populations. These were replicated three times each, and all nine simulated data sets were run through NEWHYBRIDS in parallel using the R package *parallelnewhybrid* (Wringe 2016) with 10,000 burn-in iterations and 50,000 Markov chain iterations with Jeffreys prior probabilities. Hybrid power was then assessed using the *hybridpower()* function in *hybriddetective* to determine the ability of the outlier loci to assign individuals to each of the six genotype categories. Following these simulations, we ran the outlier SNP data set through NEWHYBRIDS using the same parameters as the simulations.

**Results**

*Hybrid detection*

Based on simulated data, the power to detect hybrids in our dataset was sufficient to identify hybrids but not to detect hybrid class (F1, F2, or backcross). Using the sum of probability that an individual belongs to any hybrid class, we identified any individuals with a probably of 0.9 or higher as hybrids (Table S6). The percentage of a population sample identified as hybrids (as opposed to pure North or pure South individuals) ranged from 0 to 54.55, with NTS and SSM showing the highest levels of hybridization and samples from near the Bay of Fundy (BOF, GMI, and SSB) showing no hybridization.

**Literature Cited**

Wringe, B., I. R. Bradbury, N. W. Jeffery, and R. R. E. Stanley. *hybriddetective* in prep. Available from https://github.com/bwringe/hybriddetective.

Wringe, B. (2016) parallelnewybrid: an R package for the parallelization of hybrid detection using NEWHYBRIDS. Available from <https://github.com/bwringe/parallelnewhybrid>. DOI: 10.5281/zenodo.51281.

| Table S1. (A) Parameter values for 8 sets tested using the program *stacks* (Catchen et al. 2011) (B) Initial catalog tags, catalog tags and SNPs following initial individual filtering, and catalog tags, SNPs, number of individuals, and number of populations included following detailed filtering for missing data and minor allele frequency for each parameter set tested. The final parameter map used was 4B. | | | | | | | | |
| --- | --- | --- | --- | --- | --- | --- | --- | --- |
| (A) |  |  |  |  |  |  |  |  |
| *Stacks parameter set* | *Min. stack depth* | *Differences between stacks (1 individual)* | *Differences between secondary reads and existing stacks* | *Block haplotypes from secondary reads* | *Remove repetitive stacks* | *Deleveraging* | *Distance allowed between catalog loci* | *SNP Model* |
|  | **m** | **M** | **N** | **H** | **r** | **d** | **n** |  |
| **1** | 3 | 4 | 6 | no | yes | on | 2 | snp |
| **2** | 10 | 4 | 0 | no | yes | off | 2 | snp |
| **3** | 5 | 4 | 0 | no | yes | on | 6 | snp |
| **4** | 5 | 4 | 6 | yes | yes | on | 6 | snp |
| **4B** | 5 | 4 | 6 | no | yes | on | 6 | snp |
| **5** | 5 | 4 | 6 | yes | yes | on | 6 | bounded |
| **6** | 3 | 6 | 8 | yes | yes | on | 8 | snp |
| **7** | 3 | 4 | 6 | no | yes | off | 2 | snp |
| (B) |  |  |  |  |  |  |  |  |
| *Stacks parameter set* | *Initial catalog* | *Tags present in 75% of individuals* | | *Genotyped in 95% of individuals, MAF > 0.05, Missing loci per individual < 20%* | | |  |  |
|  | **RAD Tags** | **RAD Tags** | **SNPs** | **SNPs** | **Individuals** | **Populations** |  |  |
| **1** | 236093 | 20366 | 176492 | 6194 | 255 | 12 |  |  |
| **2** | 111104 | 16634 | 131291 | 6456 | 214 | 11 |  |  |
| **3** | 132184 | 19672 | 171923 | 7705 | 247 | 12 |  |  |
| **4** | 132281 | 19659 | 169866 | 7208 | 245 | 12 |  |  |
| **4B** | 131897 | 19672 | 173482 | 7216 | 245 | 12 |  |  |
| **5** | 132425 | 19676 | 171673 | 7516 | 246 | 12 |  |  |
| **6** | 205367 | 19945 | 182112 | 6286 | 255 | 12 |  |  |
| **7** | 230352 | 19883 | 175547 | 7157 | 255 | 12 |  |  |

| Table S2. Ten datasets used in population structure analysis of 12 populations of *P. magellanicus* collected from the Northwest Atlantic. | | | |
| --- | --- | --- | --- |
| Dataset Number | Dataset Name | Loci Included | Populations Included |
| 1 | All loci, all | All loci | SUN, LTB, MGD, NTS, PSB, BOF,  SSM, GMI, SSB, GMO, GEO, MDA |
| 2 | Outlier loci, all | Outlier loci | SUN, LTB, MGD, NTS, PSB, BOF,  SSM, GMI, SSB, GMO, GEO, MDA |
| 3 | Neutral loci, all | Neutral loci | SUN, LTB, MGD, NTS, PSB, BOF,  SSM, GMI, SSB, GMO, GEO, MDA |
| 4 | All loci, North | All loci | SUN, LTB, MGD, NTS |
| 5 | Outlier loci, North | Outlier loci | SUN, LTB, MGD, NTS |
| 6 | Neutral loci, North | Neutral loci | SUN, LTB, MGD, NTS |
| 7 | All loci, South | All loci | PSB, BOF, SSM, GMI, SSB, GMO, GEO, MDA |
| 8 | Outlier loci, South | Outlier loci | PSB, BOF, SSM, GMI, SSB, GMO, GEO, MDA |
| 9 | Neutral loci, South | Neutral loci | PSB, BOF, SSM, GMI, SSB, GMO, GEO, MDA |
| 10 | High *F*_ST_, South | 100 highest *F*_ST_ | PSB, BOF, SSM, GMI, SSB, GMO, GEO, MDA |

| **Table S3.** Comparison of outlier SNP loci from 12 populations of P. magellanicus determined using BayeScan (Bayesian method, 112 loci) and the 95% confidence intervals (72 loci) in Arlequin (hierarchical island model). Q-values were set at 0.05 in both cases.  ¤ Loci not present in Arlequin 95% * Loci not present in BayeScan | |
| --- | --- |
| Locus Name | |
| BayeScan | Arlequin 95% |
| 11_6 | 11_6 |
| 18_69¤ | 319_43 |
| 319_43 | 979_75 |
| 979_75 | 1299_57 |
| 1089_23¤ | 3619_79 |
| 1299_57 | 3834_87 |
| 1467_19¤ | 3929_9 |
| 3299_78¤ | 3969_43 |
| 3350_55¤ | 4484_19 |
| 3350_66¤ | 4668_81 |
| 3497_39¤ | 4847_58 |
| 3498_66¤ | 4975_9 |
| 3619_79 | 4975_23 |
| 3834_87 | 4975_68 |
| 3929_9 | 5252_37 |
| 3969_43 | 5439_27 |
| 4484_19 | 5750_64 |
| 4668_81 | 7326_20 |
| 4847_58 | 7396_78 |
| 4975_9 | 7524_34 |
| 4975_23 | 8699_63 |
| 4975_68 | 9554_49 |
| 5246_66¤ | 9978_28 |
| 5252_37 | 10498_47 |
| 5439_27 | 10832_30 |
| 5515_42¤ | 10832_46 |
| 5750_64 | 10832_74 |
| 5791_60¤ | 10832_84 |
| 6948_62¤ | 10987_36 |
| 7115_30¤ | 10987_48 |
| 7115_35¤ | 11531_7 |
| 7203_45¤ | 11895_12* |
| 7326_20 | 11895_17* |
| 7396_78 | 11895_50* |
| 7524_34 | 12073_7 |
| 7740_53¤ | 12308_45 |
| 8699_63 | 12767_68 |
| 8782_41¤ | 13384_35 |
| 9206_48¤ | 13891_65 |
| 9554_49 | 14394_27 |
| 9580_74¤ | 14571_64 |
| 9976_51¤ | 14750_60 |
| 9976_52¤ | 15645_89 |
| 9978_28 | 15660_9 |
| 10349_43¤ | 15821_29 |
| 10366_19¤ | 16110_74 |
| 10498_47 | 16228_63 |
| 10832_30 | 16309_22 |
| 10832_46 | 17567_80 |
| 10832_74 | 18135_35 |
| 10832_84 | 18391_26 |
| 10964_88¤ | 18656_70* |
| 10964_89¤ | 18669_56 |
| 10964_90¤ | 20298_73 |
| 10987_36 | 20400_32 |
| 10987_48 | 20633_88 |
| 11110_18¤ | 20810_86 |
| 11162_70¤ | 21297_65 |
| 11531_7 | 22237_35 |
| 12073_7 | 24442_62 |
| 12308_45 | 25322_42 |
| 12767_68 | 25380_52 |
| 13384_35 | 25380_58 |
| 13891_65 | 25380_84 |
| 14394_27 | 25405_78 |
| 14571_64 | 25627_51 |
| 14750_60 | 25881_17 |
| 15099_34¤ | 25888_33 |
| 15446_21¤ | 25888_38 |
| 15645_89 | 25962_11 |
| 15660_9 | 26519_27 |
| 15821_29 | 26726_84 |
| 16110_74 |  |
| 16228_63 |  |
| 16229_44¤ |  |
| 16309_22 |  |
| 16478_29¤ |  |
| 16894_73¤ |  |
| 17085_50¤ |  |
| 17567_80 |  |
| 18135_35 |  |
| 18391_26 |  |
| 18669_56 |  |
| 18848_34¤ |  |
| 18848_38¤ |  |
| 19165_26¤ |  |
| 19165_90¤ |  |
| 20298_73 |  |
| 20400_32 |  |
| 20548_19¤ |  |
| 20633_88 |  |
| 20810_86 |  |
| 21297_65 |  |
| 21510_19¤ |  |
| 22068_17¤ |  |
| 22237_35 |  |
| 23947_78¤ |  |
| 24442_62 |  |
| 25322_42 |  |
| 25380_52 |  |
| 25380_58 |  |
| 25380_84 |  |
| 25405_78 |  |
| 25627_51 |  |
| 25748_78¤ |  |
| 25881_17 |  |
| 25888_33 |  |
| 25888_38 |  |
| 25962_11 |  |
| 26519_27 |  |
| 26611_88¤ |  |
| 26726_84 |  |

| Table S4. Pairwise population-specific *F*_ST_ (above diagonal) and p-value (below diagonal) for (A) all loci, (B) neutral loci, and (C) outlier loci. Maximum *F*_ST_ values are highlighted in bold, minimum values are highlighted in bold and italicized. Significant p-values are italicized. | | | | | | | | | | | | |
| --- | --- | --- | --- | --- | --- | --- | --- | --- | --- | --- | --- | --- |
| (A) | **SUN** | **LTB** | **MGD** | **NTS** | **PSB** | **BOF** | **SSM** | **GMI** | **SSB** | **GMO** | **GEO** | **MDA** |
| **SUN** |  | 0.010 | 0.003 | 0.005 | 0.005 | 0.010 | 0.006 | 0.005 | 0.009 | 0.006 | 0.010 | 0.007 |
| **LTB** | *0.000* |  | 0.011 | 0.014 | 0.014 | 0.016 | 0.015 | 0.016 | 0.017 | 0.014 | **0.018** | 0.015 |
| **MGD** | *0.001* | *0.000* |  | 0.002 | 0.004 | 0.006 | 0.006 | 0.005 | 0.007 | 0.004 | 0.007 | 0.006 |
| **NTS** | *0.000* | *0.000* | *0.035* |  | 0.004 | 0.009 | 0.007 | 0.005 | 0.009 | 0.004 | 0.009 | 0.007 |
| **PSB** | *0.002* | *0.000* | 0.230 | 0.119 |  | -0.003 | -0.001 | 0.004 | -0.002 | -0.003 | 0.002 | -0.002 |
| **BOF** | *0.000* | *0.000* | *0.000* | *0.000* | 1.000 |  | 0.003 | -0.003 | 0.001 | 0.001 | 0.001 | 0.000 |
| **SSM** | *0.000* | *0.000* | *0.000* | *0.000* | 1.000 | *0.004* |  | 0.000 | 0.003 | 0.002 | 0.003 | 0.002 |
| **GMI** | *0.000* | *0.000* | *0.000* | *0.000* | 0.198 | 1.000 | 0.992 |  | -0.003 | -0.002 | -0.001 | ***-0.004*** |
| **SSB** | *0.000* | *0.000* | *0.000* | *0.000* | 1.000 | 0.672 | *0.020* | 1.000 |  | 0.001 | 0.000 | 0.000 |
| **GMO** | *0.000* | *0.000* | *0.000* | *0.000* | 1.000 | 0.629 | 0.130 | 1.000 | 0.699 |  | 0.000 | 0.000 |
| **GEO** | *0.000* | *0.000* | *0.000* | *0.000* | 0.881 | 0.687 | *0.012* | 1.000 | 0.959 | 0.965 |  | 0.000 |
| **MDA** | *0.000* | *0.000* | *0.000* | *0.000* | 1.000 | 0.916 | 0.303 | 1.000 | 0.981 | 0.996 | 0.991 |  |
|  |  |  |  |  |  |  |  |  |  |  |  |  |
| (B) | **SUN** | **LTB** | **MGD** | **NTS** | **PSB** | **BOF** | **SSM** | **GMI** | **SSB** | **GMO** | **GEO** | **MDA** |
| **SUN** |  | 0.009 | ***0.002*** | 0.003 | 0.003 | 0.007 | 0.005 | ***0.002*** | 0.005 | 0.004 | 0.005 | 0.007 |
| **LTB** | *0.000* |  | 0.010 | 0.012 | 0.011 | 0.012 | 0.013 | 0.012 | 0.012 | 0.011 | 0.012 | **0.014** |
| **MGD** | *0.040* | *0.000* |  | 0.003 | 0.002 | 0.004 | 0.005 | 0.003 | 0.005 | 0.003 | 0.004 | 0.005 |
| **NTS** | 0.073 | *0.000* | 0.640 |  | 0.002 | ***-0.004*** | -0.002 | 0.004 | -0.002 | -0.003 | -0.003 | 0.001 |
| **PSB** | *0.001* | *0.000* | 0.238 | 0.743 |  | 0.006 | 0.006 | 0.002 | 0.006 | 0.002 | 0.004 | 0.006 |
| **BOF** | *0.000* | *0.000* | *0.000* | 1.000 | *0.000* |  | 0.002 | -0.003 | 0.001 | 0.001 | 0.000 | 0.001 |
| **SSM** | *0.000* | *0.000* | *0.000* | 1.000 | *0.000* | 0.090 |  | -0.001 | 0.002 | 0.002 | 0.001 | 0.002 |
| **GMI** | *0.006* | *0.000* | *0.007* | 0.316 | 0.060 | 1.000 | 0.999 |  | ***-0.004*** | -0.002 | ***-0.004*** | -0.001 |
| **SSB** | *0.000* | *0.000* | *0.000* | 1.000 | *0.000* | 0.820 | 0.423 | 1.000 |  | 0.000 | 0.000 | 0.000 |
| **GMO** | *0.000* | *0.000* | *0.009* | 1.000 | *0.016* | 0.689 | 0.363 | 1.000 | 0.880 |  | -0.001 | 0.000 |
| **GEO** | *0.000* | *0.000* | *0.000* | 1.000 | *0.000* | 0.954 | 0.775 | 1.000 | 0.987 | 0.998 |  | 0.000 |
| **MDA** | *0.000* | *0.000* | *0.000* | 0.968 | *0.000* | 0.753 | 0.199 | 1.000 | 0.977 | 0.979 | 0.993 |  |
|  |  |  |  |  |  |  |  |  |  |  |  |  |
| (C) | **SUN** | **LTB** | **MGD** | **NTS** | **PSB** | **BOF** | **SSM** | **GMI** | **SSB** | **GMO** | **GEO** | **MDA** |
| **SUN** |  | 0.057 | 0.122 | 0.061 | 0.095 | 0.122 | 0.175 | 0.157 | 0.187 | 0.152 | 0.123 | 0.171 |
| **LTB** | *0.000* |  | 0.171 | 0.080 | 0.148 | 0.177 | 0.229 | 0.225 | **0.253** | 0.216 | 0.160 | 0.220 |
| **MGD** | *0.000* | *0.000* |  | 0.047 | 0.109 | 0.129 | 0.147 | 0.148 | 0.195 | 0.158 | 0.110 | 0.172 |
| **NTS** | *0.000* | *0.000* | *0.000* |  | 0.073 | 0.086 | 0.135 | 0.120 | 0.165 | 0.128 | 0.079 | 0.142 |
| **PSB** | *0.000* | *0.000* | *0.000* | *0.000* |  | 0.049 | 0.024 | 0.029 | 0.057 | 0.041 | 0.008 | 0.046 |
| **BOF** | *0.000* | *0.000* | *0.000* | *0.000* | *0.000* |  | 0.065 | 0.040 | 0.086 | 0.057 | 0.037 | 0.072 |
| **SSM** | *0.000* | *0.000* | *0.000* | *0.000* | *0.011* | *0.000* |  | 0.012 | 0.019 | 0.011 | 0.008 | 0.010 |
| **GMI** | *0.000* | *0.000* | *0.000* | *0.000* | *0.001* | *0.000* | *0.035* |  | 0.022 | 0.015 | 0.013 | 0.007 |
| **SSB** | *0.000* | *0.000* | *0.000* | *0.000* | *0.000* | *0.000* | *0.003* | *0.001* |  | 0.006 | 0.026 | 0.012 |
| **GMO** | *0.000* | *0.000* | *0.000* | *0.000* | *0.000* | *0.000* | *0.049* | *0.009* | 0.160 |  | 0.007 | ***0.003*** |
| **GEO** | *0.000* | *0.000* | *0.000* | *0.000* | 0.221 | *0.000* | 0.115 | *0.023* | *0.001* | 0.115 |  | 0.007 |
| **MDA** | *0.000* | *0.000* | *0.000* | *0.000* | *0.001* | *0.000* | *0.086* | 0.127 | *0.046* | 0.334 | 0.152 |  |

| Table S5. Optimal number of genetic clusters (*K*) in 12 populations of *P. magellanicus* found by several analytical methods using 10 datasets listed in Table 3. K clusters indicated by * were suggested by the analysis, but no clear clustering could be observed. | | | |
| --- | --- | --- | --- |
| Dataset Number | Dataset Description | Clustering Method | Number of clusters |
| 1 | All loci, all | Bayesian clustering, Structure | 2 |
| 2 | Outlier loci, all | Bayesian clustering, Structure | 2 |
| 3 | Neutral loci, all | Bayesian clustering, Structure | 2 |
| 4 | All loci, North | Bayesian clustering, Structure | 2 |
| 5 | Outlier loci, North | Bayesian clustering, Structure | 2 |
| 6 | Neutral loci, North | Bayesian clustering, Structure | 2 |
| 7 | All loci, South | Bayesian clustering, Structure | 6* |
| 8 | Outlier loci, South | Bayesian clustering, Structure | 2* |
| 9 | Neutral loci, South | Bayesian clustering, Structure | 4* |
| 10 | High *F*_ST_, South | Bayesian clustering, Structure | 2* |
| 1 | All loci, all | k-means clustering, Principal components analysis, adegenet | 1 |
| 2 | Outlier loci, all | k-means clustering, Principal components analysis, adegenet | 4 |
| 3 | Neutral loci, all | k-means clustering, Principal components analysis, adegenet | 1 |

| Table S6. Number of sampled individuals, number of detected hybrids (F1, F2, or backcross), and percentage of population identified as hybrid for 12 populations of *P. magellanicus*. | | | |
| --- | --- | --- | --- |
| **Population** | **Number of sampled individuals** | **Number of hybrids detected** | **Hybrids per population sample (%)** |
| SUN | 20 | 3 | 15.00 |
| LTB | 21 | 2 | 9.52 |
| MGD | 21 | 6 | 28.57 |
| NTS | 22 | 12 | 54.55 |
| PSB | 12 | 4 | 33.33 |
| BOF | 22 | 0 | 0.00 |
| SSM | 19 | 10 | 52.63 |
| GMI | 20 | 0 | 0.00 |
| SSB | 22 | 0 | 0.00 |
| GMO | 22 | 4 | 18.18 |
| GEO | 22 | 1 | 4.55 |
| MDA | 22 | 2 | 9.09 |

| Table S7. Estimates of the standard deviation of parent-offspring dispersal distance (in km) of *P. magellanicus* in (A) all populations, (B) north populations, and (C) south populations using all loci, neutral loci, and outlier loci, and low and high estimates of adult density calculated using the slope of the isolation-by-distance (IBD) relationship between pairwise population *F*_ST_ and pairwise population current-based distances and least cost distances (*marmap*). Significance of the IBD relationship is indicated as follows: p < 0.05 = *, p < 0.01 = **, p < 0.001 = ***. | | | | |
| --- | --- | --- | --- | --- |
| (A) |  |  |  |  |
| Loci included | Current | | Least Cost | |
|  | Low adult density | High adult density | Low adult density | High adult density |
| All loci | 3.8111 ± 3.1294 * | 1.6084 ± 0.7374 * | 2.8502 ± 2.3404 *** | 2.1506 ± 0.9860 *** |
| Neutral loci | 5.5712 ± 4.5746 | 2.3993 ± 1.1000 | 4.2519 ± 3.4913 | 3.1438 ± 1.4412 |
| Outlier loci | 0.9506 ± 0.7805 ** | 0.3807 ± 0.1746 ** | 0.6747 ± 0.5540 *** | 0.5364 ± 0.2459 *** |
|  |  |  |  |  |
| (B) |  |  |  |  |
| Loci included | Current | | Least Cost | |
|  | Low adult density | High adult density | Low adult density | High adult density |
| All loci | 12.9331 ± 10.6196 | 7.2980 ± 3.3459 | 3.0842 ± 2.5325 | 1.7404 ± 0.7979 |
| Neutral loci | 10.9543 ± 8.9948 | 6.1814 ± 2.8339 | 3.6747 ± 3.0174 | 2.0736 ± 0.9507 |
| Outlier loci | 1.2141 ± 0.9969 | 0.6851 ± 0.3141 | 0.5597 ± 0.4596* | 0.3158 ± 0.1448* |
|  |  |  |  |  |
| (C ) |  |  |  |  |
| Loci included | Current | | Least Cost | |
|  | Low adult density | High adult density | Low adult density | High adult density |
| All loci | 3.8111 ± 3.2194 | 2.1506 ± 0.9860 | 7.2589 ± 5.9604 | 4.0961 ± 1.8779 |
| Neutral loci | 5.5712 ± 4.5746 | 3.1438 ± 1.4413 | 5.6093 ± 4.6059 | 3.1652 ± 1.4511 |
| Outlier loci | 0.9506 ± 0.7805 | 0.5364 ± 0.2459 | 1.7271 ± 1.4182 | 0.9746 ± 0.4468 |


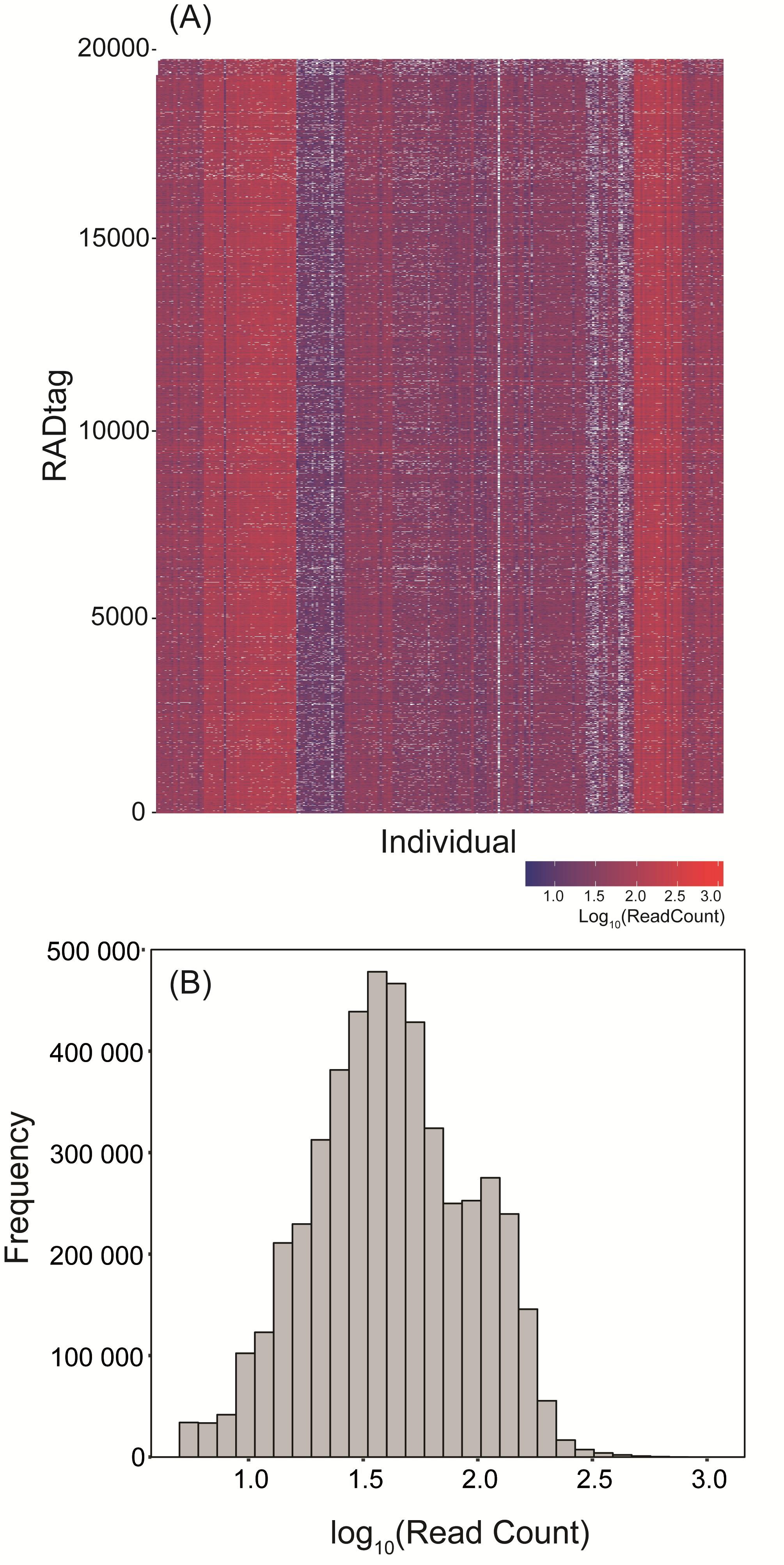


Figure S1. (A) Heat map of log_10_(ReadCount) for each individual / RADtag following initial filtering to retain RADtags present in 75% of individuals. (B) Histogram of the frequency distribution of each log_10_(ReadCount) for each individual / RADtag.


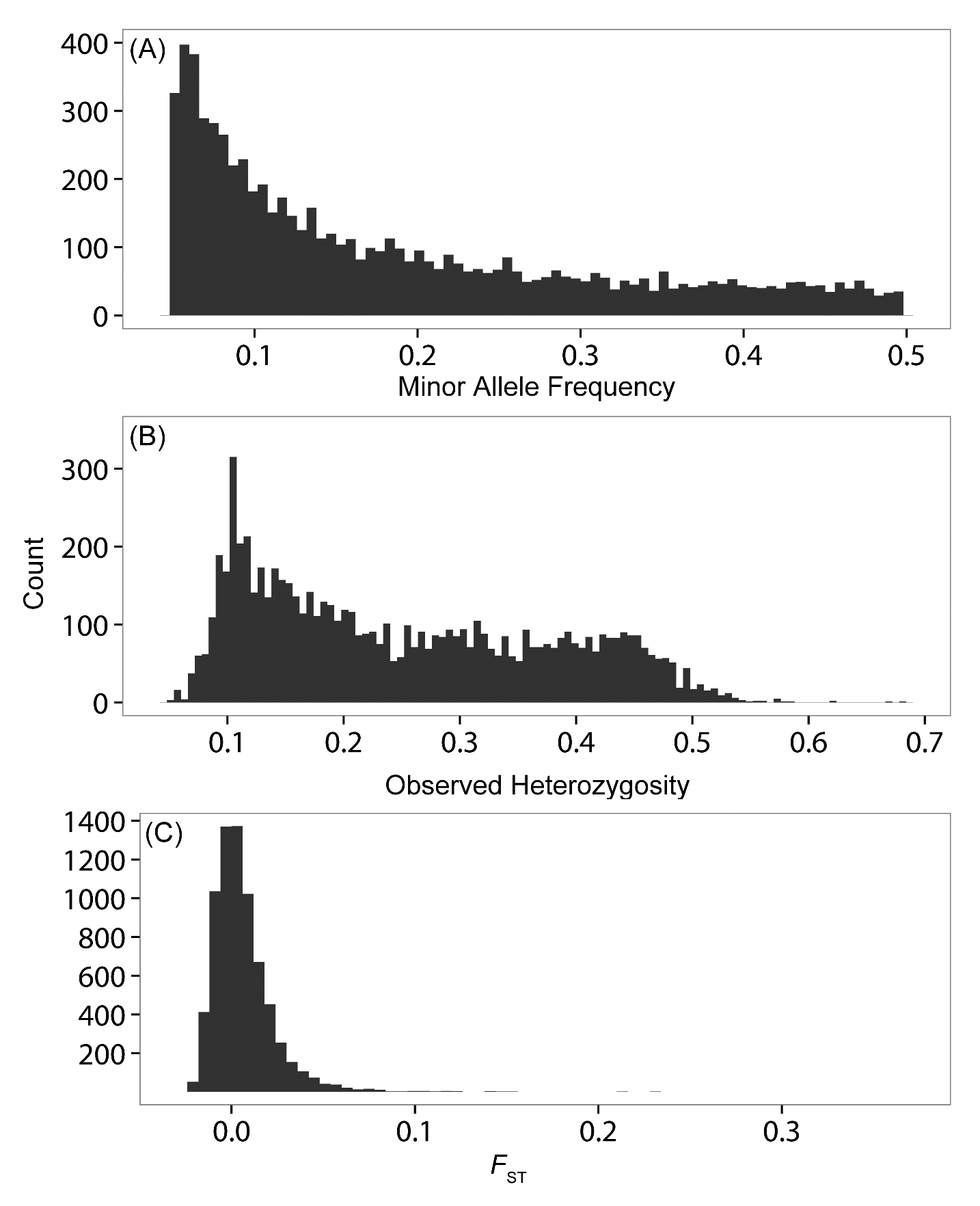


Figure S2. Histogram of minor allele frequencies, observed heterozygosity, *F*_ST_ for 7163 SNP loci sequenced in 245 adult *P. magellanicus*.


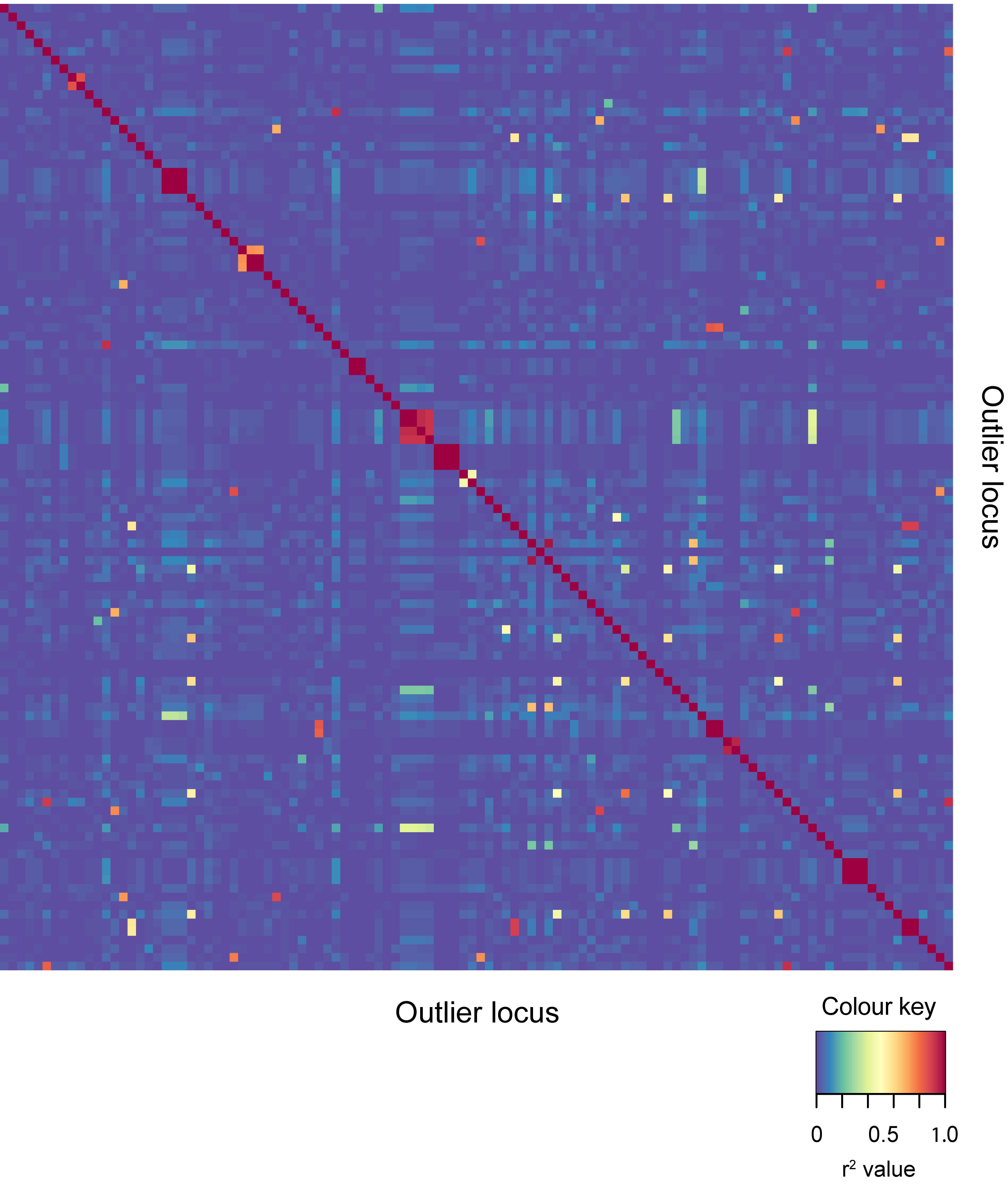


Figure S3. Heatmap of pairwise linkage disequilibrium r^2^ values calculated for 112 outlier loci detected among 12 populations of *P. magellanicus*.


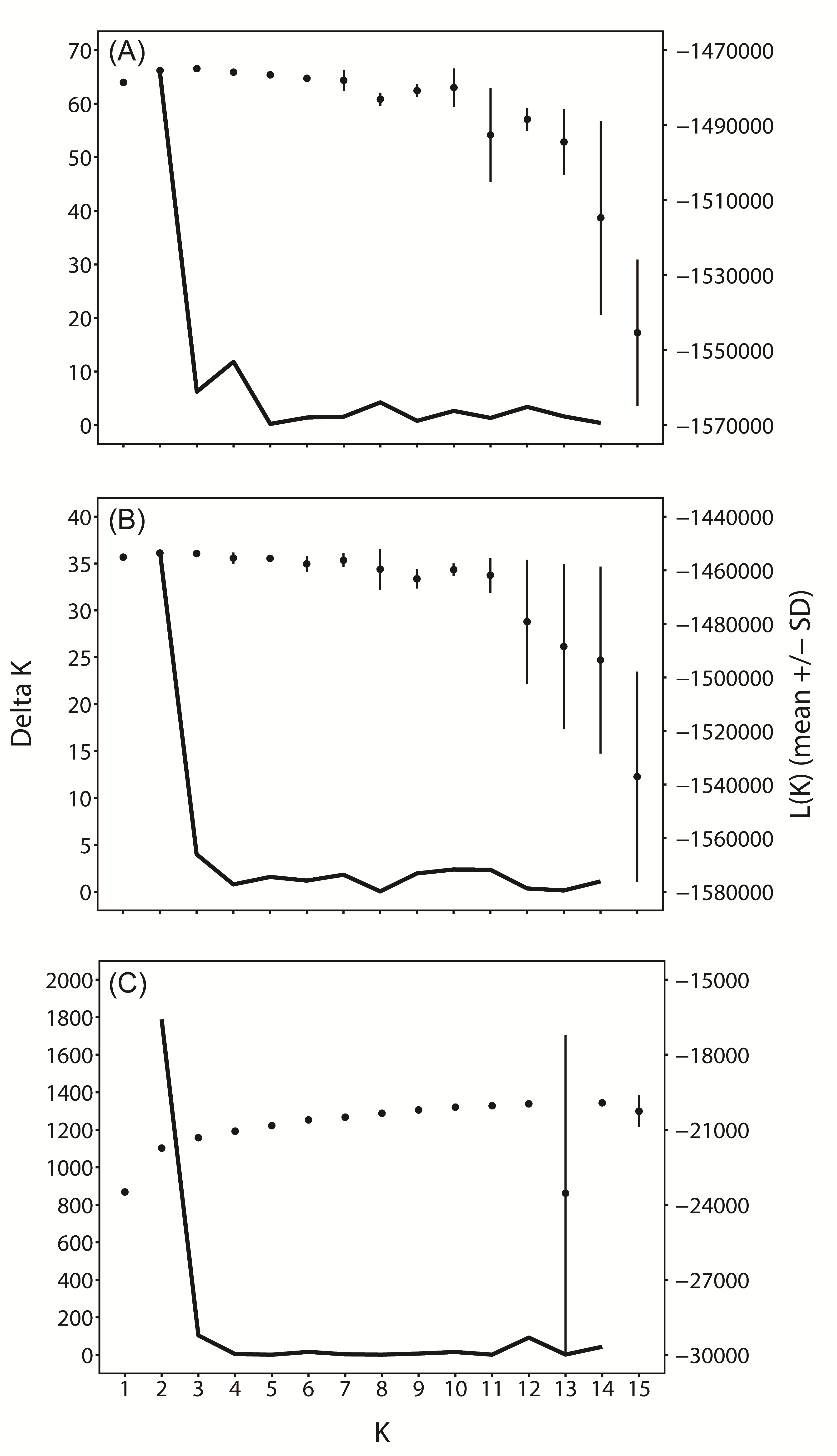


Figure S4. Delta *K* (solid line) and Ln(*K*) determined by Bayesian clustering implemented in the program Structure for 12 populations of *P. magellanicus* using (a) all loci, (b) neutral loci, and (c) outlier loci for *K* = 1-15.


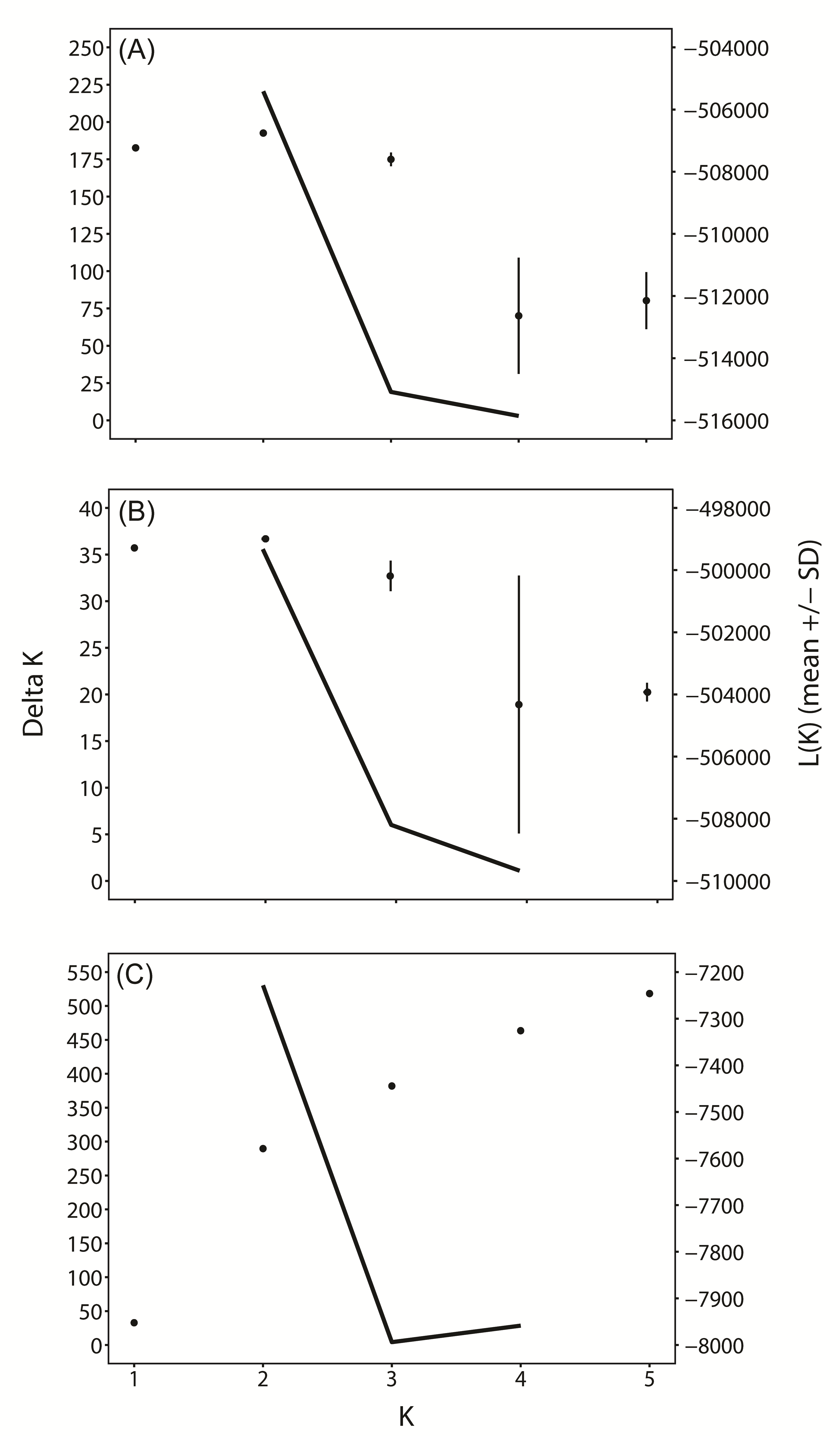


Figure S5. Delta *K* (solid line) and Ln(*K*) determined by Bayesian clustering implemented in the program Structure for 4 north populations of *P. magellanicus* using (a) all loci, (b) neutral loci, and (c) outlier loci for *K* = 1-5.


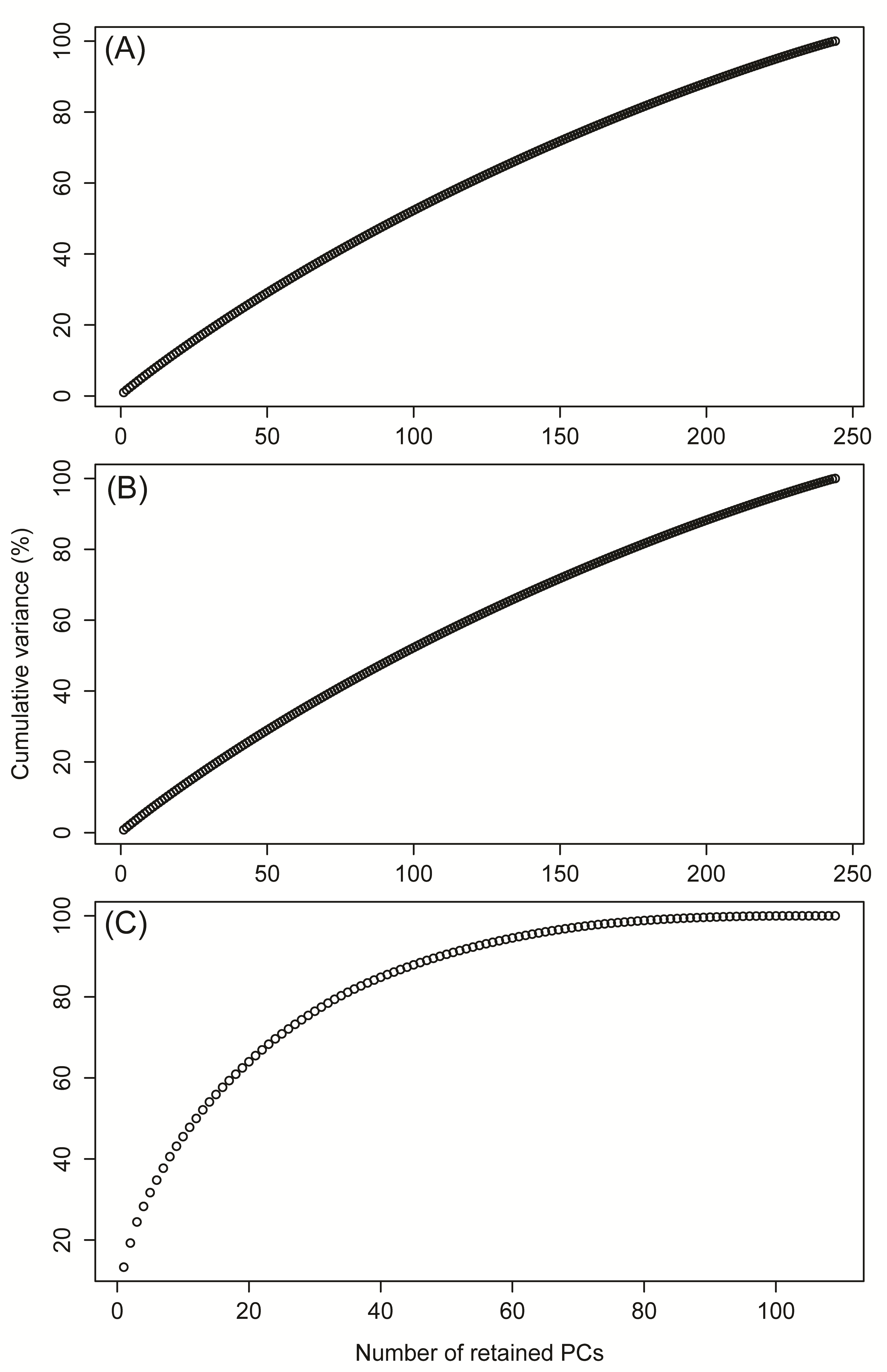


Figure S6. Cumulative variance explained by the principal components of a principal components analysis of 12 populations of *P. magellanicus* using (a) all loci, (b) neutral loci, and (c) outlier loci.


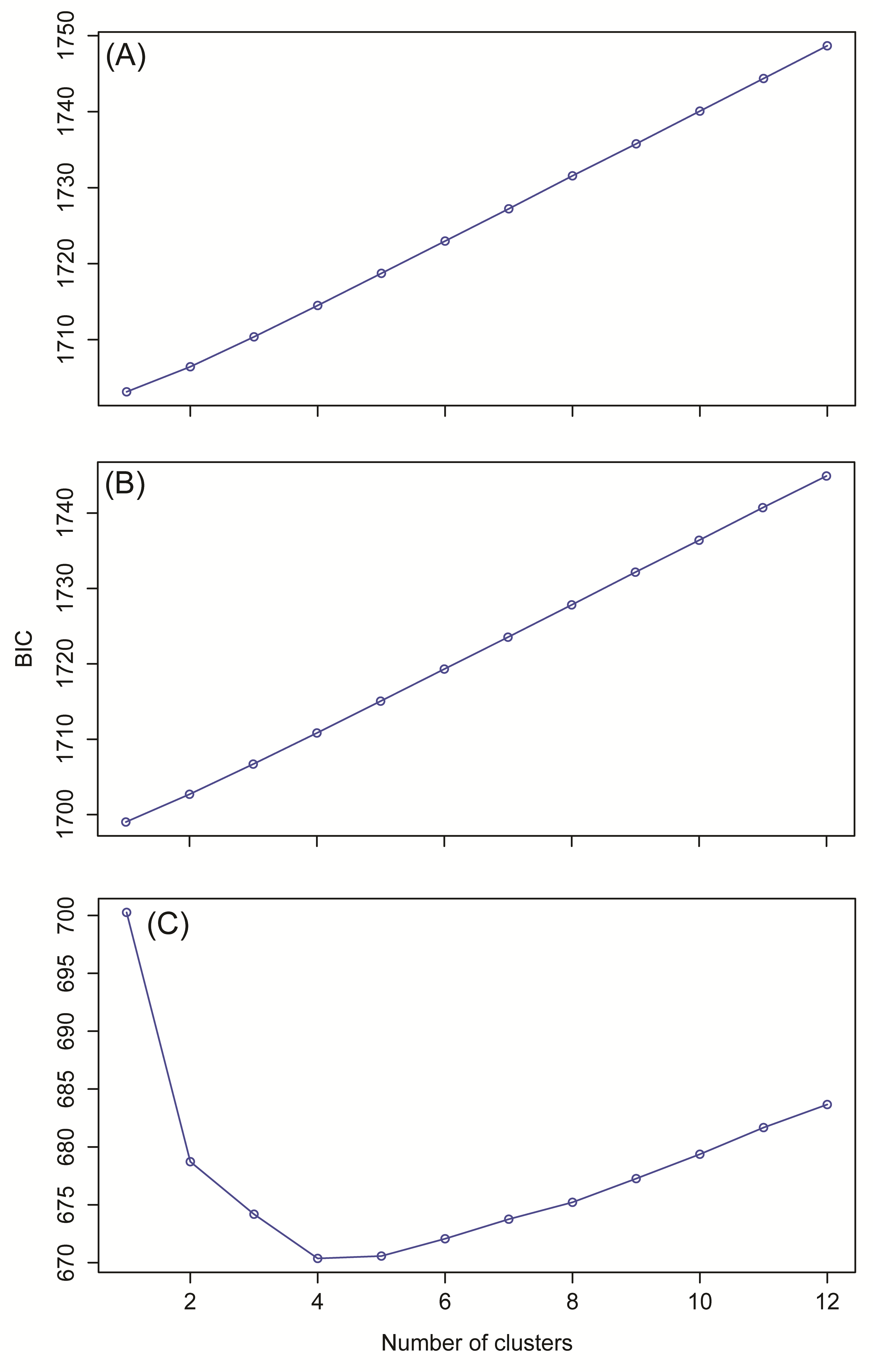


Figure S7. Bayesian Information Criterion values generated from *k*-means clustering of principal components of 12 populations of *P. magellanicus* for (a) all loci, (b) neutral loci, and (c) outlier loci.


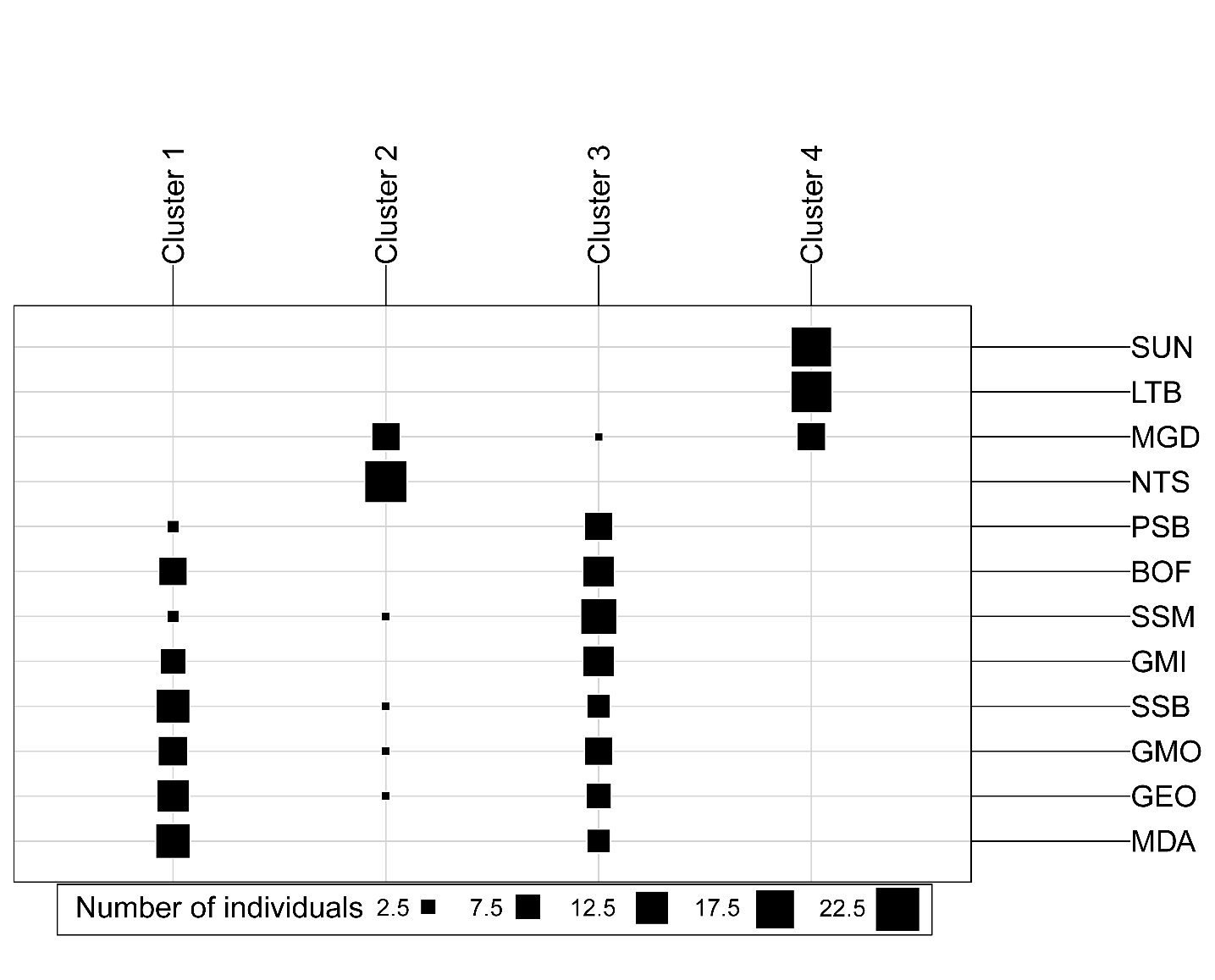


Figure S8. Plot showing the individual-specific membership of 12 *P. magellanicus* populations in each of 4 genetic clusters identified by *k*-means clustering on the principal components analysis of the outlier loci. Square size corresponds to the number of individuals from each population assigned to a particular cluster.
